# Young glial progenitor cells competitively replace aged and diseased human glia in the adult chimeric mouse brain

**DOI:** 10.1038/s41587-023-01798-5

**Published:** 2023-07-17

**Authors:** Ricardo Vieira, John N. Mariani, Nguyen P. T. Huynh, Hans J. T. Stephensen, Renee Solly, Ashley Tate, Steven Schanz, Natasha Cotrupi, Marzieh Mousaei, Jon Sporring, Abdellatif Benraiss, Steven A. Goldman

**Affiliations:** 1 Center for Translational Neuromedicine, University of Copenhagen Faculty of Health and Medical Sciences, Copenhagen, Denmark; 2 Center for Translational Neuromedicine, University of Rochester Medical Center, Rochester, NY USA; 3 Sana Biotechnology, Inc, Cambridge, MA USA; 4 Department of Computer Science, University of Copenhagen Faculty of Science, Copenhagen, Denmark

**Keywords:** Glial stem cells, Astrocyte

## Abstract

Competition among adult brain cells has not been extensively researched. To investigate whether healthy glia can outcompete diseased human glia in the adult forebrain, we engrafted wild-type (WT) human glial progenitor cells (hGPCs) produced from human embryonic stem cells into the striata of adult mice that had been neonatally chimerized with mutant Huntingtin (*mHTT*)-expressing hGPCs. The WT hGPCs outcompeted and ultimately eliminated their human Huntington’s disease (HD) counterparts, repopulating the host striata with healthy glia. Single-cell RNA sequencing revealed that WT hGPCs acquired a YAP1/MYC/E2F-defined dominant competitor phenotype upon interaction with the host HD glia. WT hGPCs also outcompeted older resident isogenic WT cells that had been transplanted neonatally, suggesting that competitive success depended primarily on the relative ages of competing populations, rather than on the presence of *mHTT*. These data indicate that aged and diseased human glia may be broadly replaced in adult brain by younger healthy hGPCs, suggesting a therapeutic strategy for the replacement of aged and diseased human glia.

## Abstract (web summary)

Old or diseased human glial cells in the chimeric mouse brain are eliminated by transplanted young cells.

## Main

Glial dysfunction is a causal contributor to a broad spectrum of neurological conditions. Astrocytic and oligodendrocytic pathologies have been associated with the genesis and progression of a number of both neurodegenerative and neuropsychiatric disorders, including conditions as varied as amyotrophic lateral sclerosis^1–4^, Huntington’s disease (HD)^5–10^ and Parkinson’s disease^11,12^, as well as schizophrenia and bipolar disease^13–19^. In such conditions, the replacement of diseased glia by healthy human glial progenitor cells (hGPCs) might provide real therapeutic benefit^20^, given their ability to disperse and colonize their hosts while giving rise to new astrocytes and oligodendrocytes. Yet, while human GPCs can outcompete and replace their mouse counterparts in a variety of experimental therapeutic models^21–23^, it has remained unclear if allografted human GPCs can replace other human cells, diseased or otherwise. Here we used human glial chimeric mice^24^ to model competition between healthy and diseased human glia in vivo, by engrafting healthy hGPCs into the striata of adult mice neonatally chimerized with hGPCs derived from participants with HD. HD is a prototypic monogenic neurodegenerative disease, resulting from the expression of a mutant, CAG-repeat expanded, mutant Huntingtin (*mHTT*) gene^25^. We had previously established that glial pathology is causally involved in the synaptic dysfunction of HD and that replacement of *mHTT*-expressing mouse glia by implanted healthy hGPCs was sufficient to both delay disease progression and rescue important elements of function in transgenic HD mouse models^5^. On that basis, in this study, we used genetically tagged wild-type (WT) and *mHTT*-expressing hGPCs, derived from sibling lines of human embryonic stem cells (hESCs), to ask if healthy WT hGPCs can replace diseased HD hGPCs in vivo. We found that when healthy hGPCs were delivered into the striata of adult mice chimerized with HD hGPCs, the healthy hGPCs outcompeted and displaced the already-resident HD hGPCs. However, because the WT donor cells were effectively younger than the resident host glia that they were replacing, we asked if differences in cell age might also contribute to competitive outcomes. We found this to be so, in that healthy young hGPCs implanted into adult mice that had been neonatally engrafted with separately tagged glia derived from the same healthy line, inexorably replaced their older isogenic counterparts. Single-cell RNA-sequencing (scRNA-seq) analysis of the younger winning and older losing hGPC populations revealed a set of differentially expressed pathways that overlapped those of winning WT and losing HD hGPCs, suggesting a common transcriptional signature of competitively dominant hGPCs. These data indicate that dynamic competition among clonally distinct glial populations may occur in the mature adult brain and that the replacement of both existing and diseased glia may thereby be achieved by the introduction of young healthy hGPCs.

## Generation of distinctly color-tagged human glia from WT and HD hESCs

**Extended Data Fig. 1:**
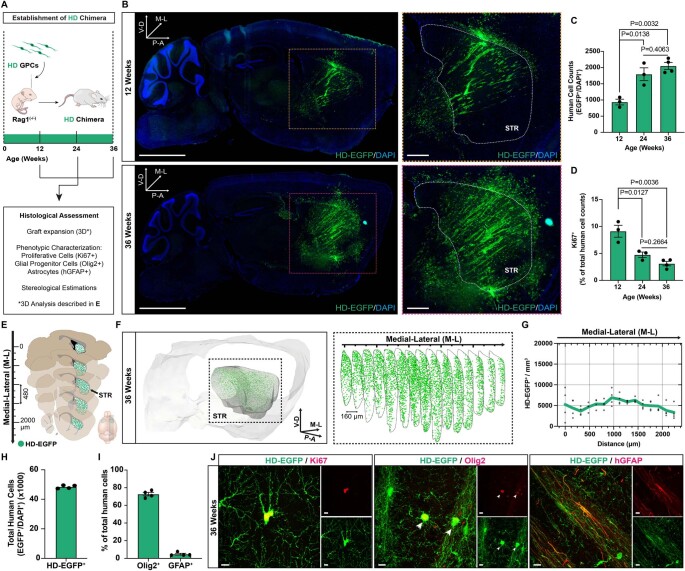
Generation of human HD glial chimeric striata. **a**. Experimental design and analytical endpoints. **b**. Neonatally engrafted HD glia (EGFP+, green) expand within the murine striatum yielding substantial humanization of the tissue over time. Images are representative of n = 3 independent experiments at 12 weeks, and n = 4 at 36 weeks. Dashed lines demarcate the striatal borders within which human cells were mapped and quantified. **c**,**d**. Their expansion is concomitant with an increase in the number of HD glia harbored in the murine striatum over time (**c**) at the cost of their Ki67+ proliferative cell pool (**d**). **e**. Strategy employed to assess the extent of striatal humanization 36 weeks following neonatal implantation of HD GPCs. HD cell distribution was mapped in 15 equidistant sagittal sections (5 are shown for example) and reconstructed in 3D for analysis. **f**. Rendered example of a mapped and reconstructed striatum for volumetric analysis. **g**. Volumetric quantification shows that by 36 weeks HD glia had expanded throughout whole striatum assuming a uniform distribution; Data are shown as mean (line) and individual data points (n = 4). **h**–**j**. As they colonized the murine striatum, HD glia either expanded and persisted as Olig2+ GPCs (arrows point to Olig2+/EGFP+ (red/green) cells) or differentiated into hGFAP+ (red) astrocytes. Proliferating (Ki67+, red) HD glia can be found even after 36 weeks of expansion, albeit in decreased numbers (**d**). STR, neostriatum; LV, lateral ventricle; CTX, cortex. **c**,**d**; **h-i**: Data presented as means ± s.e.m. with individual data points (n = 4). **c**,**d**. One-way ANOVA with Tukey’s multiple comparisons test; 12 weeks (n = 3), 24 weeks (n = 3), 36 weeks (n = 4). Scale: **b**, 500 µm; **h**, 10 µm. Source data

To assess the ability of healthy glia to replace their diseased counterparts in vivo, we first generated fluorophore-tagged reporter lines of WT and HD hESC, so as to enable the production of spectrally distinct GPCs of each genotype, whose growth in vivo could then be independently monitored. We first used a CRISPR–Cas9-mediated knock-in strategy^26^ to integrate EGFP and mCherry reporter cassettes into the AAVS1 locus of matched, female sibling WT (GENEA019) and *mHTT*-expressing (HD, GENEA020) hESCs^27,28^ (Supplementary Fig. 1). We then verified that the reporter cassettes stably integrated into each of these clones (Supplementary Fig. 2a) and that editing did not influence the self-renewal, pluripotency or karyotypic stability of the tagged hESCs (Supplementary Fig. 2b,c). From these tagged and spectrally distinct lines, we used our previously described differentiation protocol^5^ to produce color-coded human glial progenitor cells (hGPCs) from each line, whose behaviors in vivo could be compared, both alone and in competition. We validated the ability of each line to maintain EGFP or mCherry expression after maturation as astrocytes or oligodendrocytes, and their lack of any significant differentially expressed oncogenic mutations, or copy number variants (CNVs) that could bias growth (Supplementary Fig. 2d,e); we also verified that both the WT and *mHTT*-expressing hGPCs, when injected alone, colonized the mouse host brains (Extended Data Figs. 1a,b and 3a,b).

We then differentiated both WT-mCherry and HD-EGFP hESCs using our established protocol for generating hGPCs^21^ and assessed both their capacity to differentiate into glia and the stability of their reporter expression upon acquisition of glial fate (Supplementary Fig. 3a–d). By 150 days in vitro, glial cultures derived from both WT-mCherry and HD-EGFP were equally enriched for PDGFRα^+^/CD44^+^ bipotential GPCs^29^ (*P* = 0.78), comprising around half of the cells in the cultures, with the rest being immature A2B5^+^ GPCs^30^ and PDGFRα^−^/CD44^+^ astrocytes and their progenitors^31^ (Supplementary Figs. 3c and 4a,b). Notably, virtually all immuno-phenotyped cells derived from WT-mCherry and HD-EGFP hESCs—including mature astrocytes as well as hGPCs—continued to express their respective fluorescent reporter, indicating that transgene expression remained stable upon acquisition of terminal glial identity, both in vitro (Supplementary Fig. 3d) and after subsequent transplantation in vivo (Supplementary Fig. 5).

## Establishment of human HD glial chimeric mice

Using these spectrally distinct WT and HD hGPCs, we first asked if resident *mHTT*-expressing HD glia were less fit and hence potentially replaceable by their healthy counterparts. To this end, we generated mice whose striata were substantially chimerized by tagged *mHTT*-expressing glia, by neonatally injecting hGPCs derived from EGFP-tagged HD hESCs into the neostriata of immunodeficient *Rag1*^*−/−*^ mice (Extended Data Fig. 1a). Following implantation, the HD glia rapidly infiltrated the striata of these mice, migrating and expanding first within the striatal white matter tracts and then progressively displacing their mouse counterparts from the striatal neuropil (Extended Data Fig. 1b). As a result, by 36 weeks, the mouse striatum was substantially humanized by HD glia (Extended Data Fig. 1b,f,g). The colonization of the host striatum by human HD GPCs was driven by the mitotic expansion of the HD hGPCs, the total number of which typically more than doubled between 12 weeks and 36 weeks (Extended Data Fig. 1c; *P* = 0.0032). In contrast, as these cells achieved their terminal densities in their hosts, their proliferative cell pool (Ki67^+^) progressively declined (Extended Data Fig. 1d; *P* = 0.0036), resulting in their slowed expansion rate over time.

Most HD glia expanded as Olig2^+^ GPCs (72.7 ± 1.9%), which persisted as the new resident pool after replacing their mouse counterparts. A fraction of these (4.8 ± 0.9%) further differentiated into GFAP^+^ astrocytes (Extended Data Fig. 1i,j). Astrocytic differentiation was mostly observed within striatal white matter tracts. These sick astrocytes lacked the structural complexity typically observed in healthy counterparts and displayed abnormal fiber architecture as previously reported^8^ (Extended Data Fig. 1j).

## Healthy hGPCs infiltrate the HD chimeric striatum to replace host glia

**Fig. 1:**
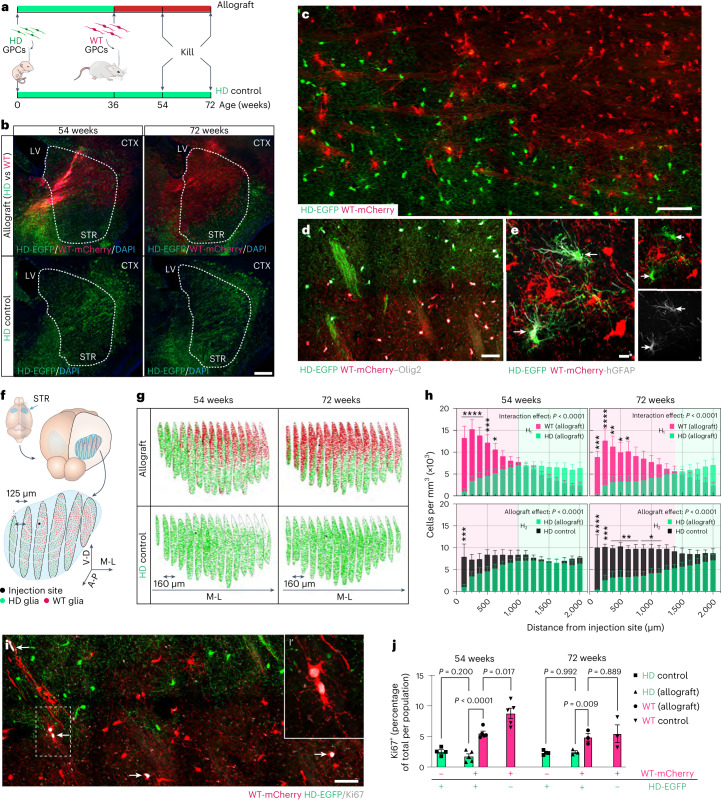
Adult-transplanted WT human GPCs outcompete and replace neonatally resident HD hGPCs. **a**, Experimental design and analytical endpoints. **b**, Engraftment of WT glia (mCherry^+^, red) into the striatum of HD chimeras yielded progressive replacement of HD glia (EGFP^+^, green). Images are representative of *n* = 8 independent experiments at 54 weeks, *n* = 7 at 72 weeks for allograft groups and *n* = 4 at each timepoint for HD control groups. Dashed outlines (white) demarcate the striatal outlines within which human cells were scored. **c**, The border between advancing WT (mCherry, red) and retreating HD (EGFP, green) hGPCs was typically well delineated. **d**, Competing WT and HD cells were identified as hGPCs such by their mutual expression of Olig2 (white). Images are representative of *n* = 8 experiments. **e**, GPC replacement preceded astrocytic replacement, as within regions colonized by WT hGPCs, stray HD astrocytes (hGFAP^+^, white) were still found. Images representative of *n* = 8 experiments. **f**, Mapped distributions of human glia in host striata. Human glia was mapped in 15 equidistant sections (five shown as examples) and reconstructed in 3D. Their distribution was measured radially as a function of distance to the injection site. **g**, Rendered examples of mapped striata of both adult allografted (top) and HD-only control (bottom), at 54 and 72 weeks. **h**, Volumetric quantification confirmed that WT cells replaced their HD counterparts as they expanded from their implantation site. Top row: H_1_, interaction of allografted WT versus HD cells, at 54 and 72 weeks. The advance of WT cells was accompanied by the progressive elimination of HD glia. *P* < 0.0001 at both 54 weeks (*n* = 8 mice) and 72 weeks (*n* = 7). Bottom row: H_2_, comparison of HD hGPCs exposed to WT cells (HD allograft; *n* = 8 at 54 weeks, *n* = 7 at 72 weeks) versus unchallenged HD cells (HD control; *n* = 4 at both timepoints); *P* < 0.0001 at each timepoint, two-way analysis of variance (ANOVA) with Šidák’s multiple comparisons tests. Data presented as means ± s.e.m. **i**, At the boundary between WT and HD glia, Ki67^+^ (white) cells predominate within the WT glial population. Inset i′ shows higher magnification of two WT daughter cells at the edge of the competitive boundary. **j**, Quantification of Ki67^+^ glia within each population as a function of time shows a sustained proliferative advantage by WT glia. WT versus HD (allograft), *P* < 0.0001 at 54 weeks and *P* = 0.009 at 72 weeks; HD control, 54 weeks (*n* = 4) and 72 weeks (*n* = 4); WT control, 54 weeks (*n* = 5) and 72 weeks (*n* = 3); WT versus HD allograft, 54 weeks (*n* = 5) and 72 weeks (*n* = 3). Comparisons by two-way ANOVA with Šidák’s multiple comparisons tests; means ± s.e.m. ****P < 0.0001, ***P < 0.001, **P < 0.01 and *P < 0.05. Scale bars, 500 µm (**b**), 100 µm (**c**), 50 µm (**d**), 10 µm (**e**), 100 μm (**i**), 10 μm (**i**, inset i′). CTX, cortex; LV, lateral ventricle; STR, striatum (caudate-putamen). Source data

**Extended Data Fig. 2:**
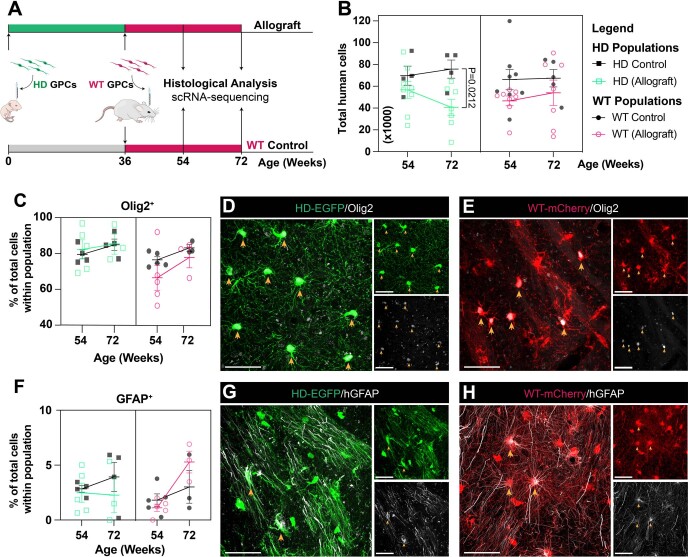
Replacement of HD by WT glial progenitor cells yields proportionate phenotypic replacement. **a**. Experimental design and analytical endpoints for the WT control group. **b**. Stereological estimations demonstrate that the total number of HD glia progressively decreases relatively to HD chimera controls as WT glia expands within the humanized striatum; 2-way ANOVA with Šidák’s multiple comparisons test. HD control: n = 4 for both timepoints; WT control: n = 5 for 54 wks, n = 3 for 72 wks; allograft: n = 5 for 54 wks, n = 3 for 72 wks. **c**–**e**. WT glia expanded as Olig2+ (white) GPCs displacing their HD counterparts. **f**–**h**. Within areas where they became dominant, they further differentiated into hGFAP+ (white) astrocytes. The proportion of GPCs and astrocytes in both populations was maintained as they competed for striatal dominance. Orange arrows point to co-labeled cells. **c–h**: HD control: n = 4 for both timepoints; WT control: n = 5 for 54 wks, n = 3 for 72 wks; Allograft: n = 5 for 54 wks, n = 3 for 72 wks. **b**-**c**, **f**: Data shown as means ± SEMs with individual data points. Scale: **d-e**, **g-h**, 50 µm. Source data

Having established chimeras whose striatal glia were largely *mHTT*-expressing and human, we next asked how these resident HD human glia might respond to the introduction of healthy hGPCs. To that end, we engrafted hGPCs derived from WT hESCs engineered to express mCherry into the striata of 36-week-old HD chimeras and monitored their expansion histologically as they competed with the already-resident HD glia (Fig. 1a and Extended Data Figs. 2a,b).

Following engraftment, the WT glia pervaded the previously humanized striatum, gradually displacing their HD counterparts as they expanded from their implantation site (Fig. 1b). This process was slow but sustained, over time yielding substantial repopulation of the HD striatum with WT glia (Fig. 1b,g; H_1_: 54 weeks, *P* < 0.0001; 72 weeks, *P* < 0.0001). Remarkably, the expansion of WT glia was paralleled by a concurrent elimination of HD glia from the tissue (Fig. 1b,g; H_2_: 54 weeks, *P* < 0.0001; 72 weeks, *P* < 0.0001). This was typically characterized by a discrete advancing front, behind which few HD glia could be found (Fig. 1c). As a result, mutually exclusive domains were formed in the wake of this competition between HD and WT GPCs (Fig. 1d). Of note, within regions dominated by WT glia, we found GFAP-defined HD astrocytes to often linger, primarily within white matter tracts (Fig. 1e). Astrocytic replacement progressed more slowly than did that of hGPCs, so that when assessed at 72 weeks, most WT donor cells still expressed Olig2^+^ (80.1 ± 4.7%), while only a fraction (4.0 ± 1.5%) had differentiated as GFAP^+^ astrocytes (Extended Data Fig. 2c–h). As such, astrocytic replacement appeared to depend upon astrocytic turnover with replacement from the locally dominant parenchymal hGPCs, rather than from competition among mature astrocytes; dynamic competition within the time frame studied appeared to be primarily a feature of hGPCs.

**Extended Data Fig. 3:**
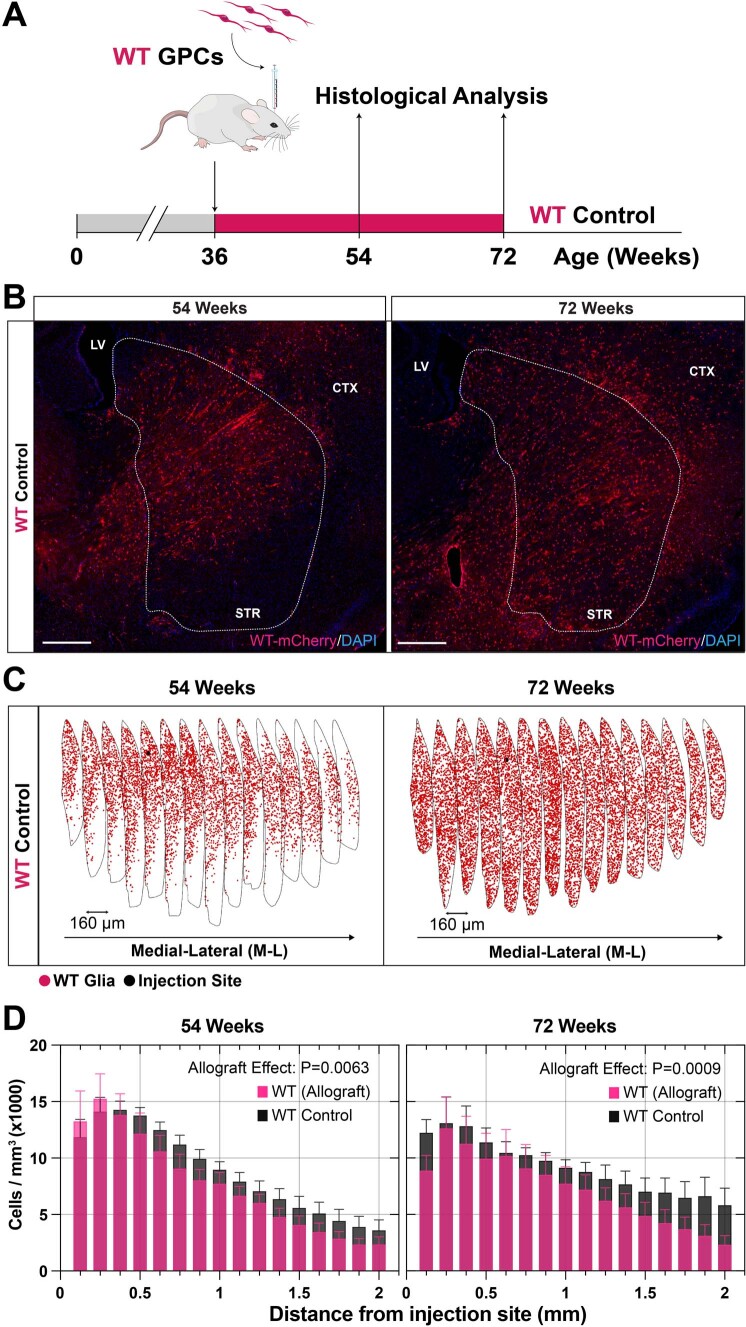
Adult-engrafted hGPCs more rapidly dominate already-resident mouse than human hGPCs. **a**. Experimental design and analytical endpoints for the WT control group. **b**. Engraftment of WT glia (mCherry+, red) into the striatum of adult Rag1−/− mice yields substantial humanization of the murine striatum over time. Images are representative of n = 8 independent experiments for the 54 week timepoint, and n = 5 for 72 weeks. Dashed outlines demarcate the striatal borders within which human cells were mapped and quantified. **c,d**. Volumetric quantifications show that adult-transplanted WT control glia infiltrate and disperse throughout the murine striatum over time (**c**), and (**d**), and that they do so significantly more broadly than do those WT allograft cells transplanted into already HD chimeric mice. WT allografts (into HD chimeras; data comparison to Fig. 2): n = 8 for 54 weeks, n = 7 for 72 weeks; WT controls: n = 8 for 54 weeks, n = 5 for 72 weeks. **d**: Two-way ANOVA with Šidák’s multiple comparisons test; main effects are shown as P values; data presented as means ± SEM. Scale: **b**, 500 µm. Source data

Of note, the allogeneic replacement of hGPCs by other hGPCs proceeded at a slower rate than the xenogeneic replacement of mouse by hGPCs, as WT hGPCs implanted into naïve adult *Rag1*^*−/−*^ mice expanded throughout the host striatum more rapidly and broadly than did WT hGPCs transplanted into adult HD chimeras (Extended Data Fig. 3a–d; 54 weeks, *P* = 0.006; 72 weeks, *P* = 0.0009). These results indicate that competitive glial replacement develops with kinetics that differs between xenogeneic and allogeneic grafts, with greater—but readily surmountable—competitive resistance posed by same-species cells.

**Extended Data Fig. 4:**
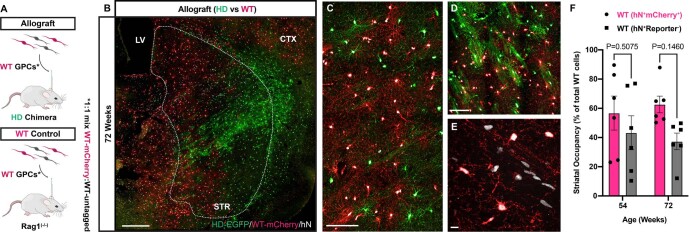
Expression of fluorescent transgenes did not influence competitive dominance. Co-engrafted isogenic clones of tagged and untagged WT hGPCs admixed while displacing resident HD glia. **a**. Experimental design for mice that received a 1:1 mixture of mCherry-tagged (WT-mCherry) and untagged (WT-untagged) WT glia. **b**. Immunolabeling against human nuclear antigen (hN) shows that both WT-mCherry (mCherry+ hN+, red, white) and WT-untagged (mCherry- EGFP- hN+, white) glia expanded within the previously humanized striatum, progressively displacing HD glia (EGFP+ hN+, green, white). **c**. Vast homotypic domains were formed as mixed WT glia expanded and displaced resident HD glia. **d**. In contrast, isogenic WT-mCherry and WT-untagged were found admixing. **e**. As previously noted, within WT glia dominated domains, only more complex astrocyte-like HD glia could be found, typically within white matter tracts. **f**. Quantification of the proportion of WT-mCherry and WT-untagged glia within the striatum showed no significant difference between the two populations at either quantified timepoint (n = 6 for each timepoint; samples were pooled from both experimental groups); Two-way ANOVA with Šidák’s multiple comparisons test; Data are shown as means ± SEM with individual data points. Scale: **b**, 500 µm; **c-d**, 100 µm; **e**, 10 µm. Source data

In parallel control studies, we established that these diverse observations were not an artifact of off-target effects of either gene editing or fluorescent reporter toxicity. Co-engrafted hGPCs derived from WT-mCherry and their unmodified counterparts (WT-untagged) expanded equally within the striata of both naïve adult *Rag1*^*−/−*^ and HD chimeric mice; the tagged and untagged WT cells, otherwise isogenic, admixed freely and yielded analogous glial repopulation in each (Extended Data Fig. 4a–d,f; 54 weeks, *P* = 0.50; 72 weeks, *P* = 0.15).

## Human WT glia enjoy a proliferative advantage over resident HD glia

**Extended Data Fig. 5:**
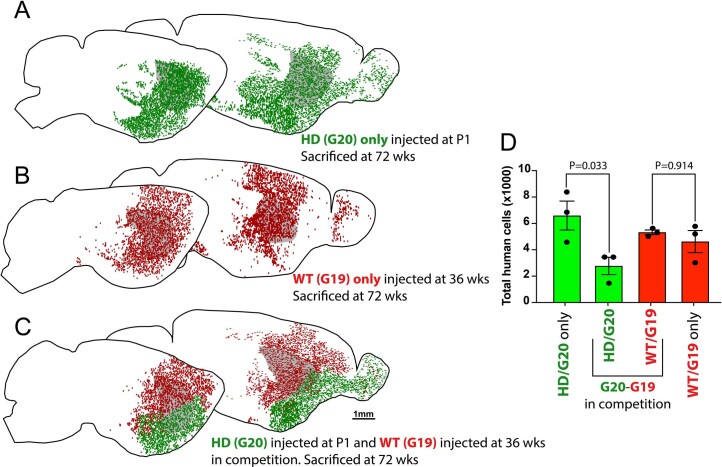
Progressive extrastriatal expansion and competitive dominance of WT over HD glial progenitors. Human GPC chimeric mice were established by either a neonatal striatal injection of HD EGFP-tagged hGPCs (**a**; G20 only), mCherry-tagged wildtype (WT) hGPCs transplanted into untreated 36 week-old mice (**b**; G19 only), or mCherry-tagged WT hGPCs transplanted into HD-EGFP neonatally-engrafted mice at 36 weeks (**c**). All mice were killed at 72 weeks, and human cells mapped in equidistant serial brain sections. In all groups, cells dispersed beyond the striatum where the cells were injected, ultimately colonizing much of the anterior forebrain. **d**. Quantification of mapped human cells. n = 3 for all experimental groups. Data shown as means ± SEMs with individual data points. *p = 0.033, by one-way ANOVA with Tukey’s post hoc test. Source data

Because striatal humanization by HD glia decelerated with time as the fraction of proliferative HD hGPCs fell (Extended Data Fig. 1d), we next asked if the selective expansion of younger WT glia within the HD striatum was sustained by a difference in proliferative capacity between the two populations. To do so, we assessed the expression of Ki67 in both WT and HD glial populations as competitive striatal repopulation unfolded. At both 54 and 72 weeks of age, the mitotic fraction of implanted WT human glia was significantly larger than that of resident HD-derived human glia (Fig. 1i,j; 54 weeks, *P* < 0.0001; 72 weeks, *P* = 0.009). These data suggest that the repopulation of the HD glial chimeric host striatum by WT glia was mediated in part via the selective expansion of a differentially proliferative donor pool. Of note, while the proliferative advantage of the adult-engrafted WT glia over the already-resident HD glia became less pronounced as the mice—and hence the cells—aged, it was maintained through at least 72 weeks of age, suggesting the persistence of WT glial competitive dominance beyond the observed experimental timepoints. Interestingly, we noted that throughout that 72-week period of observation, the leading edge of the neonatally implanted human HD glia continued to slowly expand well beyond the striatum into the basal forebrain, even as the leading edge of the adult-transplanted WT hGPCs replaced that striatal population, effectively from behind (Extended Data Fig. 5).

## Human WT glia assume a dominant competitor profile when facing HD glia

**Fig. 2:**
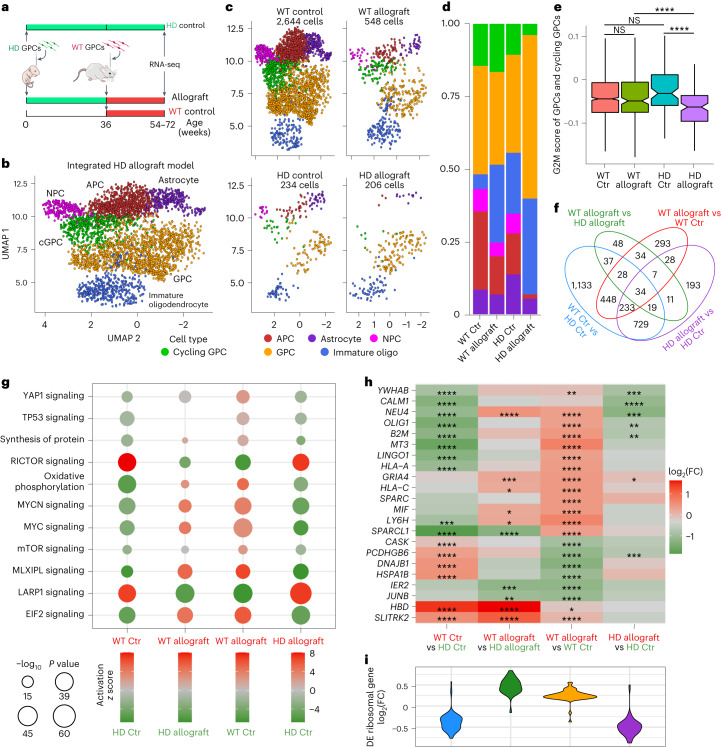
WT glia acquire a dominant competitor profile in the face of resident HD glia. **a**, Experimental design. **b**,**c**, UMAP visualization of the integrated (**b**) and split by group (**c**) scRNA-seq data identifies six major cell populations. **d**, Stacked bar plot proportions of cell types in each group. **e**, Cell cycle analysis, notched box plots of cycling GPCs and GPCs in the G2/M phase (WT Ctr, *n* = 1,371 cells; WT allograft, *n* = 266 cells; HD Ctr, *n* = 104 cells; HD allograft, *n* = 124 cells). The box indicates the interquartile range, the notch indicates the 95% confidence interval with the median at the center of the notch, and the error bars represent the minimum and maximum nonoutlier values. **f**, Venn diagram of pairwise differentially expressed GPC genes (log_2_(FC) > 0.15, adjusted *P* < 0.05, calculated using MAST and listed in Supplementary Table 3). **g**, Curated IPA of genes differentially expressed between GPC groups (listed in Supplementary Table 4). The size of circles represents *P* value, while the shading indicates activation *z* score with red being more active in the upper group and green being more active in the lower group. **h**, Heatmap of curated pairwise differentially expressed GPC genes. **i**, Violin plots of pairwise differentially expressed GPC ribosomal gene log_2_ FCs. Comparisons between groups in **e** used Dunn tests following a Kruskal–Wallis test. All tests had multiple comparisons adjusted via the Benjamini–Hochberg method. *P < 0.05, **P < 0.01, ***P < 0.001 and ****P < 0.0001. FC, fold change. Source data

Having established that implanted WT hGPCs effectively colonize the HD glial chimeric striata at the expense of the resident *mHTT*-expressing glia, we next sought to define the molecular signals underlying their competitive dominance. To that end, we analyzed the transcriptional profiles of WT and HD human glia isolated from the striata of chimeras in which the two cell populations were coresident and competing, as well as from their respective controls in which one or the other was transplanted without the other, using scRNA-seq (10X Genomics, v3.1 chemistry; Fig. 2a). Following the integration of all captures and aligning against human and mouse mixed species genome, Leiden community detection revealed six major populations of human glia, which included hGPCs, cycling hGPCs, immature oligodendrocytes, neural progenitor cells, astrocytes and their intermediate progenitors (astrocyte progenitor cells; Fig. 2b–d). Within these populations, cell cycle analysis predicted higher G2/M scores in competing WT hGPCs compared to their HD counterparts (Fig. 2e), aligning with our histological observations (Fig. 1j). To proceed, we focused on hGPCs as the primary competing population in our model. Pairwise differential expression revealed discrete sets of differentially expressed genes across groups (Fig. 2f and Supplementary Table 3), and subsequent functional analysis with Ingenuity Pathway Analysis (IPA) within the hGPC population revealed numerous salient terms pertaining to their competition (Fig. 2g and Supplementary Table 4).

We found that during competition, WT GPCs activate pathways driving protein synthesis, whereas HD GPCs were predicted to downregulate them. Predicted upstream transcription factor (TF) activation identified YAP1, MYC and MYCN—conserved master regulators of cell growth and proliferation^32–34^—as significantly modulated across experimental groups. Notably, we found YAP1 and MYC targets to be selectively downregulated in competing HD GPCs relative to their controls (Fig. 2g,h). Notably, this downregulation was attended by marked repression of ribosomal encoding genes (Fig. 2i). Conversely, competing WT hGPCs showed an upregulation of both YAP1 and MYC targets, as well as in the expression of ribosomal encoding genes, relative to controls (Fig. 2g,i). As such, these data suggest that the implanted WT hGPCs actively assumed a competitively dominant phenotype upon contact with their HD counterparts, to drive the latter’s local elimination while promoting their own expansion and colonization.

## Age differences drive competitive human glial replacement

**Fig. 3:**
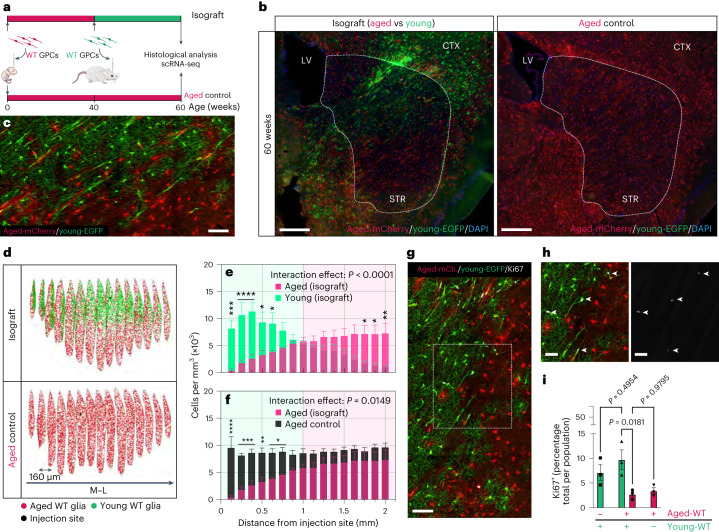
Differences in cell age are sufficient to drive competitive repopulation of humanized striata. **a**, Experimental design and analytical endpoints. **b**, Engraftment of younger WT glia (EGFP^+^, green) into the striatum of WT chimeras yielded selective replacement of their aged counterparts (mCherry^+^, red). Dashed outlines demarcate the striatal regions within which human cells were mapped and quantified. Images are representative of *n* = 3 independent experiments for each group. **c**, A higher power image of the border between younger and older cells, highlighting the narrow zone within which these populations interact as the younger cells invade. **d**, Rendered examples of mapped striata. Volumetric quantification shows that the younger WT glia replaces their older isogenic counterparts as they expand from their injection site. **e**, Aged versus young (isograft), *P* < 0.0001 (*n* = 3). Their advance tracked the progressive elimination of aged WT glia from the tissue, relative to control WT chimeras (aged control). **f**, Aged (isograft) versus aged (control) *P* = 0.0149 (*n* = 3 each); two-way ANOVA with Šidák’s multiple comparisons test. Interactions or main effects are shown as numerical *P* values, while post hoc comparisons are shown as follows: ****P < 0.0001, ***P < 0.001, **P < 0.01, *P < 0.05; data presented as means ± s.e.m. **g**,**h**, At the interface between young and aged WT glia, a higher incidence of Ki67^+^ (white) cells can be seen within the younger population. The dashed square is shown in higher power in **h** which includes a color split highlighting the Ki67^+^ cells. **i**, Quantification of Ki67^+^ cells shows that younger WT glia are significantly more proliferative than their aged counterparts; *P* = 0.018 (*n* = 3 for all groups). One-way ANOVA with Šidák’s multiple comparisons test; data are shown as means ± s.e.m with individual data points. Scale bars, 500 µm (**b**), 100 µm (**c**), 100 µm (**g**) and 50 µm (**h**). Source data

Because WT cells transplanted into adult hosts were fundamentally younger than the resident host cells that they displaced and replaced, we next asked if differences in cell age, besides disease status, might have contributed to the competitive success of the late donor cells. To that end, we engrafted hGPCs newly produced from WT hESCs engineered to express EGFP into the striata of 40-week-old adult glial chimeras, which had been perinatally engrafted with hGPCs derived from mCherry-tagged, otherwise isogenic WT hESCs (Fig. 3a). We then monitored the expansion of the transplanted cells histologically, so as to map the relative fitness and competitive performance of these isogenic, but otherwise distinctly aged pools of hGPCs.

We noted that the expansion of implanted WT glia within the striatum of WT chimeras was strikingly similar to their expansion in the striata of HD chimeras (Fig. 1). Following engraftment, the younger WT glia rapidly infiltrated the previously humanized striatum, progressively displacing their aged counterparts as they expanded from their implantation site, ultimately yielding substantial recolonization of the tissue (Fig. 3b–d,e; *P* < 0.0001). Their expansion was paralleled by the local elimination of aged WT glia (Fig. 3b–d,f; *P* < 0.0001), which was also marked by a discrete advancing front, behind which few already-resident WT glia could be found (Fig. 3c). Accordingly, we also noted that the mitotic fraction of implanted WT glia was significantly larger than that of their resident aged counterparts (Fig. 3g–i; *P* = 0.018). Together, these data indicated that the repopulation of the human WT glial chimeric striatum by younger isogenic hGPCs was attended by the replacement of the older cells by their younger counterparts, fueled in part by the relative expansion of the younger, more mitotically active cell population.

## Young hGPCs replace older cells via the induction of apoptosis

**Extended Data Fig. 6:**
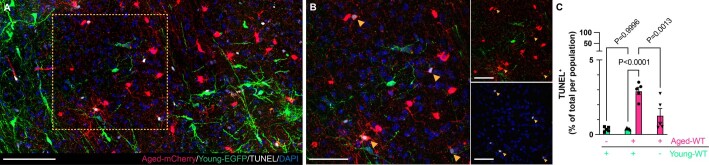
Aged human glia are eliminated by their younger counterparts through induced apoptosis. **a**. At the border between young (EGFP+, green) and aged WT glia (mCherry+, red), a higher incidence of apoptotic TUNEL+ (white) cells are apparent in the aged population. **b**. Higher magnification of a competitive interface between these distinct populations shows resident glia selectively undergoing apoptosis. **c**. Quantification of TUNEL+ cells shows significantly higher incidence of TUNEL+ cells among aged resident WT glia, relative to both their younger isogenic counterparts (P < 0.0001), and to aged WT chimeric controls not challenged with younger cells (P = 0.0013). Quantification was performed on pooled samples from 60 and 80 weeks timepoints (n = 5 for all experimental groups). One-way ANOVA with Šidák’s multiple comparisons test; data are shown as means ± SEM with individual data points. Scale: **a**, 100 µm; **b**, 50 µm. Source data

Because younger glia appeared to exert clear competitive dominance over their older counterparts, we next asked whether the elimination of the older glia by younger cells occurred passively, as a result of the higher proliferation rate of the younger cells leading to the relative attrition of the older residents during normal turnover, or whether the replacement was actively driven by the induction of programmed cell death in the older cells by the more fit younger cells. To address this question, we used the terminal deoxynucleotidyl transferase-dUTP nick end labeling (TUNEL) assay to compare the rates of apoptosis in aged and young WT glial populations as they competed in the host striatum, as well as at their respective baselines in singly transplanted controls. We found that as competitive repopulation unfolded, that aged WT glia underwent apoptosis at a markedly higher rate than their younger counterparts (Extended Data Fig. 6a–c; *P* < 0.0001). Critically, the increased apoptosis of older, resident glia appeared to be driven by their interaction with younger cells, because a significantly higher proportion of aged glia was found to be apoptotic in chimeras transplanted as adults with younger cells than in controls that did not receive the later adult injection (Extended Data Fig. 6c; *P* = 0.0013). These data suggest that aged resident glia confronted by their younger counterparts are actively eliminated, at least in part via apoptosis triggered by their encounter with the younger hGPCs, whose greater relative fitness permitted their repopulation of the chimeric host striatum.

## Young hGPCs become dominant when challenged with older isogenic cells

**Fig. 4:**
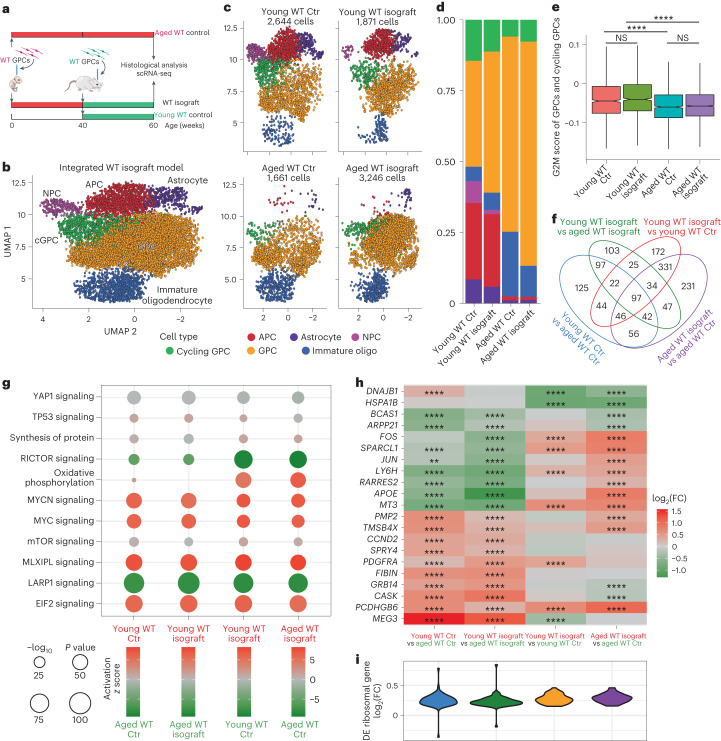
WT glia acquire a dominant transcriptional profile when confronting their aged counterparts. **a**, Experimental design. **b**,**c**, UMAP visualization of the integrated (**b**) and split by group (**c**) scRNA-seq data identifies six major cell populations. **d**, Stacked bar plot proportions of cell types in each group. **e**, Cell cycle analysis notched box plots of cycling GPCs and GPCs in the G2/M phase (young WT Ctr, *n* = 1,371 cells; young WT isograft, 1,141; aged WT Ctr, 1,242; aged WT isograft, 2,817). The box indicates the interquartile range, the notch indicates the 95% confidence interval with the median at the center of the notch and the error bars represent the minimum and maximum nonoutlier values. **f**, Venn diagram of pairwise differentially expressed GPC genes (log_2_(FC) > 0.15, adjusted *P* value < 0.05, calculated using MAST and listed in Supplementary Table 5). **g**. Curated IPA of genes differentially expressed between GPC groups (listed in Supplementary Table 6). As in Fig. 2, the size of circles represents *P* value, while the shading indicates the activation *z* score with red being more active in the upper group and green being more active in the lower group. **h**, Heatmap of curated pairwise differentially expressed GPC genes. **i**, Violin plots of pairwise differentially expressed GPC ribosomal gene log_2_ FCs. Comparisons between groups in **e** used Dunn tests, following a Kruskal–Wallis test. All tests had multiple comparisons adjusted via the Benjamini–Hochberg method. *P < 0.05, **P < 0.01, ***P < 0.001 and ****P < 0.0001. Source data

To ascertain if the molecular signals underlying the competitive dominance of younger WT glia over aged WT glia are similar to those underlying their dominance over HD glia, we analyzed the transcriptional signatures of competing young and aged WT glia and their respective controls, using scRNA-seq (Fig. 4a). Within the sequenced hGPC populations (Fig. 4b–d and see also Supplementary Fig. 6), we noted that the G2/M scores in competing aged WT cells were markedly lower than those of their younger counterparts (Fig. 4e), in accord with our histological data (Fig. 3i). Differential expression analysis revealed discrete sets of genes differentially expressed between competing young and aged WT GPCs (Fig. 4f,h and Supplementary Table 5), and subsequent IPA analysis of those gene sets revealed a signature similar to that observed between donor (young) WT and already-resident (aged) HD GPCs in our competitive allograft model (Fig. 4g and Supplementary Table 6). In particular, genes functionally associated with protein synthesis, including ribosomal genes and E2F family members, as well as upstream MYC and MYCN signaling, were all activated in competing young WT GPCs relative to their aged counterparts (Fig. 4g). Yet despite these similarities, in other respects aged WT GPCs responded differently than did HD GPCs to newly implanted WT GPCs. In contrast to HD GPCs, aged WT cells confronted with younger isogenic competitors upregulated both MYC and MYCN targets relative to their noncompeting controls (Fig. 4g,h) with a concomitant upregulation of ribosomal genes (Fig. 4i). This difference in their profiles may represent an intrinsic capacity to respond competitively when challenged, which *mHTT*-expressing HD hGPCs lack. Nonetheless, this upregulation was insufficient to match the greater fitness of their younger counterparts, which similarly—but to a relatively greater degree—manifested the selective upregulation of MYC targets, as well as ribosomal genes, relative to their noncompeting controls (Fig. 4g–i). Together, these data indicate that the determinants of relative cell fitness may be conserved across different scenarios of challenge and that the outcomes of the resultant competition are heavily influenced by the relative ages of the competing populations.

## Competitive advantage is linked to a set of TFs

**Fig. 5:**
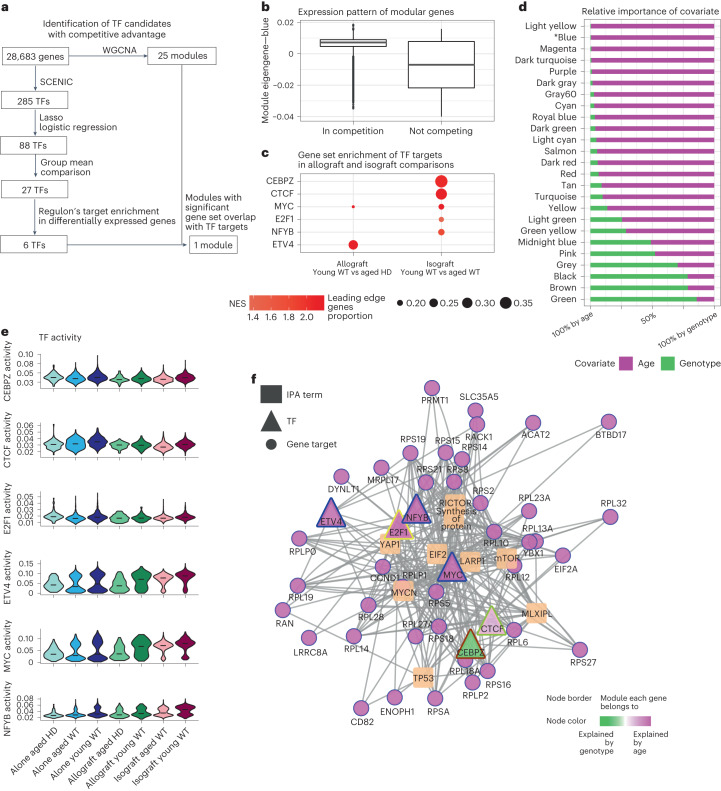
Transcriptional signature of competitive advantage. **a**, Schematic representation of our protocol for identifying TFs linked specifically to competitive advantage. **b**, Box plot of identified WGCNA module eigengene of interest (blue) in competing and noncompeting cells. Lower and upper hinges indicate the first and third quartiles (25th and 75th percentiles), with median-indicating line in the center of the box. Whiskers extend to 1.5× the interquartile range from the upper and lower hinges. **c**, Gene set enrichment analysis highlighted prioritized TFs whose regulons were enriched for upregulated genes in dominant young WT cells. **d**, Analysis of the relative contribution of each biological factor (age versus genotype) toward the composition of each module eigengene. **e**, Important TFs, as predicted by SCENIC to establish competitive advantage, and their relative activities across groups. **f**, Regulatory network including downstream targets and their functional signaling pathways. Target expressions are controlled by at least one other important TF in **e**. NES, normalized enrichment score. Source data

We next asked what gene signatures would define the competitive advantage of newly transplanted human GPCs over resident cells. To that end, we applied a multistepped analysis using Lasso-regulated logistic regression (Fig. 5a), that pinpointed six TFs (CEBPZ, CTCF, E2F1, MYC, NFYB and ETV4) whose activities could significantly explain the dominance of young WT GPCs over both aged HD and aged WT GPCs (Fig. 5e). These six TFs and their putative targets established gene sets (regulons) that were upregulated (normalized enrichment score (NES) > 0, adjusted *P* < 10^−3^) in the young WT cells, in either our allograft or isograft models (Fig. 5c). We also noticed that while their activities varied when not in a competitive environment (aged HD, aged WT and young WT alone), they were higher in the dominant young WT cells in both allograft (versus HD) and isograft (versus older isogenic self) paradigms, especially so for MYC (Fig. 5e).

Next, we set out to identify cohorts of genes with defined expression patterns, as well as significant overlaps to the six prioritized regulons above. We first employed weighted gene co-expression network analysis (WGCNA)^35^ to detect a total of 25 modules in our GPC dataset (Fig. 5a). Interestingly, only one module (blue) harbored genes with significant overlap to the targets of our TF cohort. We also noticed that genes of module blue were driven by competition, as their expression was upregulated in the competing versus noncompeting environments (Fig. 5b). We then asked if the expression pattern of prioritized modules could be explained by the age of cells (young versus old), by their genotype (HD versus WT), or both. WGCNA defines module eigengene as the first principal component of a gene cohort, representing thereby the general expression pattern of all genes within that module. As such, we built linear models where module eigengene was a response that was described by both age and genotype. We observed that module blue was primarily influenced by age (Fig. 5d), in contrast to other modules identified from WGCNA.

MYC, whose regulated pathway activation had already been inferred as conferring competitive advantage (Figs. 2 and 4), was also one of the six prioritized TFs. We, thus, further characterized the MYC regulon and its downstream targets and noticed how these downstream targets were also regulated by our other prioritized TFs (Fig. 5f). Interestingly, MYC was part of module blue and regulated these blue modular genes, whose expression levels were higher in the competing versus noncompeting paradigms (Fig. 5b), a pattern suggesting that the blue signature was not activated unless cells were in a competing environment. Furthermore, we noted lower TF activity of MYC in the aged HD relative to the aged WT hGPCs (Fig. 5e), which may highlight the intrinsically greater capacity of WT cells to compete; this was congruent with our earlier observation that aged WT hGPCs respond differently than HD hGPCs when challenged with newly engrafted WT GPCs. Notably, the blue module eigengene could be described by age, demonstrating that the competitive advantage associated with MYC signaling was driven by mostly the age of the cells. Accordingly, the targets in this network were enriched for pathways regulating cell proliferation (TP53, YAP1 and RICTOR), transcription (MYCN and MLXIPL), and protein synthesis (LARP1), each of which had been previously noted as differentially expressed in each competitive scenario (Figs. 2 and 4). The output of this competition-triggered regulatory network thus appeared to confer a competitive advantage upon young WT hGPCs when introduced into the adult brain, whether confronted by older, HD-derived or isogenic hGPCs.

## Discussion

In light of the contribution of glial pathology to a broad variety of neurodegenerative and neuropsychiatric disorders^36,37^, we sought here to establish the relative fitness of WT and both diseased and aged human GPCs in vivo, so as to assess the potential for allogeneic glial replacement as a therapeutic strategy. We initially focused on HD, given the well-described role of glial pathology in HD^5,8,10,38,39^. We found that when WT hGPCs were introduced into brains already chimerized with HD hGPCs, the WT cells outcompeted and ultimately replaced the already-resident HD glial progenitors. The selective expansion of the healthy cells was associated with the active elimination of the resident HD glia and was further supported by the proliferative advantage of the healthy donor cells relative to their already-resident diseased counterparts. scRNA-seq revealed that the dominance of healthy WT hGPCs encountering HD glia in these adult chimeric mouse brains was linked to their expression of a transcriptional signature characteristic of competitively dominant cells in invertebrate systems. Surprisingly, when we controlled for the relative ages of the already-resident (older) and newly introduced (younger) donor hGPCs, we found that WT hGPCs transplanted into adult neostriata, which had been neonatally chimerized with separately tagged but otherwise isogenic WT hGPCs, similarly outcompeted and replaced their older, already-resident counterparts. This observation suggested that cellular youth was a critical determinant of competitive success and of the ability of a donor hGPC population to replace that of the host. Accordingly, transplanted young WT hGPCs acquired the gene expression signature of a dominant competitor phenotype in vivo, whether challenged by already-resident older HD or isogenic WT hGPCs; indeed, our analysis suggested that cellular youth was an even stronger determinant of competitive fitness than was disease genotype.

These observations suggest that cell replacement was driven by a recapitulation of developmental cell competition, an evolutionarily conserved selection process by which less fit clones are sensed and eliminated from the tissue by their fitter neighbors^40–43^, but as manifested here dynamically in the adult brain. This process has been shown in a variety of systems to comprise the active elimination of relatively slowly growing cells by their faster growing, more competitively fit neighbors^44–48^. We noted that in the adult brain, WT hGPCs typically expanded from their implantation sites in an advancing proliferative wave. These younger hGPCs largely eliminated their hitherto stably resident—and hence older—counterparts, whether the latter were *mHTT*-expressing HD cells, or isogenic WT cells that had been transplanted months earlier. In both cases, the younger cells ultimately recolonized their host brains with healthy new hGPCs (Figs. 1 and 3), and in both cases, the younger donor cells differentially expressed gene sets associated with competitive dominance (Figs. 2, 4 and 5). In particular, the competitive dominance of younger, adult-transplanted hGPCs was associated with their increased levels of predicted YAP1, E2F, MYC and MYCN pathway activity. These data provided a striking parallel to cell–cell competition in the mouse embryo, in which defective cells are eliminated by their neighbors following the acquisition of differential MYC expression during competitive challenge^44,49,50^ and in which YAP1 and MYC interact to determine competitive outcomes during cell–cell competition^51,52^. Indeed, the concurrent enrichment for YAP1 pathway members in ‘winner’ WT hGPCs, including transcripts both upstream and downstream of YAP1, suggests that the Hippo pathway might be an especially promising target for the regulation of glial replacement in the adult human brain. Indeed, these observations parallel the results of liver repopulation studies, in which mouse fetal liver progenitors were found to drive faster and more extensive replacement when allografted into older than into younger hosts^53^ and for which MYC and YAP1 activities were predominant determinants of competitive success^54^. As such, the identification of YAP1 and MYC as important regulators of competition among hGPCs may enable strategies to further enhance the competitive advantage, speed and extent of donor cell colonization following the delivery of these cells to the brain.

The competitive replacement of resident glia by younger hGPCs, which we observed, resembles that of mouse glial replacement by implanted human GPCs, as their expansion within the mouse brain is also sustained by a relative proliferative advantage, and progresses with the elimination of their mouse counterparts upon contact^22^. As in the xenograft setting, the winning population of young WT hGPCs appears to trigger the apoptotic death and local elimination of the resident losing population, whether comprised of older isogenic WT or sibling HD cells. The relative localization of dying host cells to the advancing wavefronts of younger WT cells suggests that the latter trigger the death of already-resident hGPCs, likely via contact-dependent means. Potential mechanisms for such contact-dependent regulation of relative cell fitness have been described in a variety of models^40^, and include selective expression of *Fwr* isoforms^55,56^, as well as mechanical signals, potentially transduced through *Piezo1*-dependent modulation of YAP^57^. In addition, the selective elimination of both HD and isogenic hGPCs when confronted with younger hGPCs was paralleled by their depletion of ribosomal encoding transcripts, consistent with the loss of ribosomal transcripts by ‘loser’ cells during cell competition^58,59^, highlighting the contribution of ribosomal protein transcription to the regulation of cell fitness^60–63^. Together, these data suggest that the transcriptional control of translational machinery is important in cell–cell competition in the adult brain, as it is in development.

These observations suggest that the brain may be a far more dynamic structural environment than previously recognized, with cell–cell competition among glial progenitor cells—and potentially their derived astrocytes—playing a critical role in adult brain maintenance, just as in development. Indeed, the competitive advantage we noted of young over older resident cells seems to largely mimic development, where successive waves of GPCs compete among each other, with the oldest largely eradicated from the brain by birth, replaced by younger successors^64^. In adulthood, one may similarly envision that somatic mutation among dividing glial progenitors may yield selective clonal advantage to one daughter lineage or the other, resulting in the inexorable replacement of the population by descendants of the dominant daughter. This scenario, while typifying the onset of carcinogenesis, may also be involved in tumor suppression, via the competitive elimination of neoplastic cells by more fit non-neoplastic neighbors^65^. It is especially intriguing to consider whether such a process of dynamic competition among differentially fit hGPCs may be involved in the development of non-neoplastic adult-onset brain disorders in which glia are involved, such as some schizophrenias^13,14,16,17^, and HD itself^5–10^. Indeed, such a mechanism may contribute to the late-stage acceleration in disease progression often noted among those neurodegenerative and neuropsychiatric disorders in which glial pathology is involved.

These data may also have strong therapeutic implications, as they suggest that in the adult human brain, resident glia—whether diseased or simply aged—may be replaced following the introduction of younger and healthier GPCs. Given the many neurodegenerative and neuropsychiatric diseases in which causally contributory glial pathology is now recognized^1–19^, the clinical implications of this observation may be profound; it suggests that the dysfunctional glia of diseased brains, across a variety of disease etiologies and phenotypes, might be effectively eliminated and replaced by the intracerebral delivery of newly generated allogeneic hGPCs. However, it is important to recognize that these conclusions are based on our experiments in a model system that has inherent limitations. While we have endeavored to provide as humanized a model as feasible, by largely humanizing and to some extent aging the host glial population before later transplantation of younger WT human cells, these chimeric animals comprise an inherently artificial system. The time courses of both development and aging are intrinsically different for mouse and human brain cells, and the effects of a heterospecific environment on the aging process of xenografted human cells have not hitherto been defined. In addition, the vascular bed of the chimeras is that of their mouse host, and it is entirely unknown whether the migratory support afforded to hGPCs by mouse and human vascular beds might differ. Along the same vein, the host microglial cells of these chimeras are untethered to peripheral immune interaction, as these are immunodeficient hosts; this too might affect donor cell colonization in the chimeras. With these and other considerations in mind, it seems apparent that until human GPCs are transplanted into patients, we cannot be entirely sure that glial replacement will proceed as robustly as in our chimeric model. Yet notwithstanding these caveats, our results suggest that glial progenitor cell delivery and glial replacement may offer a viable and broadly applicable strategy toward the cell-based treatment of those diseases of the brain in which glial cells are causally involved.

## Methods

### hESC lines and culture conditions

Sibling hESC lines GENEA019 (WT: 18;15 CAG) and GENEA020 (HD: 48;17 CAG), both female, were obtained from Genea Biocells. hESCs were regularly cultured under feeder-free conditions on 0.55 µg cm^−2^ human recombinant laminin 521 (Biolamina, LN521) coated cell culture flasks with mTeSR1 medium (StemCell Technologies, 85850). Daily medium changes were performed. hESCs were routinely passaged at 80% confluency onto freshly coated flasks. Passaging was performed using ReLeSR (StemCell Technologies, 05872). All hESCs and differentiated cultures were maintained in a 5% CO_2_ incubator at 37 °C and routinely checked for contamination and mycoplasma-free status. The karyotypes of the source lines, both before and after reporter insertion (see ‘Generation of fluorescent reporter hESCs’), were analyzed on metaphase spreads by G-banding (Institut für Medizinishche Genetik und Angewandte Genomik, Universitätsklinikum Tübingen). All hESC lines had a normal karyotype. Additionally, acquired CNVs and loss-of-heterozygosity regions were assessed by array comparative genomic hybridization (aCGH, Cell Line Genetics; Supplementary Fig. 2). No CNVs were noted that were either known or might be expected to influence the outcome of competitive interactions between the clones.

### Generation of fluorescent reporter hESCs

For ubiquitous and distinct fluorescence labeling of WT and HD cells (Supplementary Fig. 1), reporter constructs driving expression of either mCherry or EGFP were inserted into the AAVS1 safe-harbor locus of WT GENEA019 and HD GENEA020 hESCs using a modified version of the CRISPR–Cas9-mediated strategy previously described in ref. ^26^. To prepare hESCs for plasmid delivery by electroporation, hESC were collected as single-cell suspension following dissociation with Accutase (StemCell Technologies, 07920), washed in culture medium and counted with the automated cell counter NucleoCounter NC-200 (ChemoMetec). Per electroporation, a total of 1.5 × 10^6^ cells were mixed with 5 µg of the AAVS1 targeting CRISPR–Cas9 plasmid (pXAT2) and 5 µg of reporter donor plasmid (pAAVS1-P-CAG-mCherry or pAAVS1-P-CAG-EGFP). pXAT2 (Addgene plasmid, 80494), pAAVS1-P-CAG-mCherry (Addgene plasmid, 80491) and pAAVS1-P-CAG-EGFP (Addgene plasmid, 80492) were a gift from K. Woltjen. Electroporation was performed using an Amaxa 4D-Nucleofector (Lonza) with the P3 primary cell kit (Lonza, V4XP-3024) according to the manufacturer’s guidelines. After nucleofection, the electroporated hESC suspensions were transferred to 10 cm cell culture dishes and cultured with mTeSR1 supplemented with 10 µM Y-27632 (Tocris, 1254) for the first 24 h. Electroporated hESCs were grown for 48–72 h, then treated with 0,5 µg µl^−1^ puromycin (Thermo Fisher Scientific, A1113803). Electroporated hESC cultures were kept under puromycin until individual colonies were large enough to be picked manually. Colonies were assessed by fluorescence microscopy and transferred to a 96-well plate based on the uniformity of reporter expression. Following expansion, each clone was split for further expansion and genotyping. DNA was extracted using the prepGEM tissue DNA extraction kit (Zygem) for genotyping. Correctly targeted transgenic integrations in the AAVS1 locus were detected by PCR using the following primers: dna803, 5′-TCGACTTCCCCTCTTCCGATG-3′ and dna804, 5′-CTCAGGTTCTGGGAGAGGGTAG-3′, while the zygosity of the integrations was determined by the presence or absence of a WT allele using an additional primer, which is as follows: dna803 and dna183, 5′-GAGCCTAGGGCCGGGATTCTC-3′. hESCs with correctly targeted insertions were cryopreserved with Pro-Freeze CDM (Lonza, BEBP12-769E) and then expanded for karyotype and aCGH before hGPC production.

### Derivation of hGPCs from reporter WT and HD hESCs

Human GPCs were derived from both reporter WT and HD hESCs using our described protocol^21^, with minor modifications to the embryoid body generation step (Supplementary Information). Cells were collected for transplant between 150 days and 200 days in vitro, at which time the cultures derived from both WT-mCherry/EGFP and HD-EGFP hESCs were comprised predominantly of PDGFRα^+^/CD44^+^ bipotential GPCs. See Supplementary Fig. 3 for a detailed immunocytochemical characterization of the cultures and Supplementary Fig. 4 for flow cytometric quantification, the protocols for which are included in the Supplementary Information.

### Xenotransplantation

#### Cell preparation

To prepare cells for transplant, glial cultures were collected in Ca^2+^/Mg^2+^-free Hanks’ balanced salt solution (HBSS^−/−^; Thermo Fisher Scientific, 14170112), then mechanically dissociated to small clusters by gentle pipetting and counted with a hemocytometer. The cell suspension was then spun and resuspended in cold HBSS^−/−^ at 10^5^ cells per µl, and kept on ice until transplanted.

#### Neonatal grafts

To generate human–mouse chimeras harboring *mHTT*-expressing human glia (HD chimeras), newborn immunocompromised *Rag1*^*−/−*^ pups^66^ were cryoanesthetized, secured in a custom baked clay stage and injected bilaterally with 100,000 HD glia (50,000 per hemisphere) into the presumptive striatum within 48 h of birth. Cells were delivered using a 10 μl syringe (Hamilton, 7653-01) with pulled glass pipettes at a depth of 1.2–1.4 mm. The pups were then returned to their mother until weaned.

#### Adult grafts

To assess the capacity of implanted healthy human glia to replace their diseased counterparts, 36-week-old HD glial chimeras were anesthetized by ketamine/xylazine and secured in a stereotaxic frame. In total, 200,000 WT glia were delivered bilaterally using a 10 μl syringe and metal needle into the striatum (anteroposterior (A-P), +0.8 mm; mediolateral (M-L), ±1.8 mm; dorsoventral (D-V), −2.5 to −2.8 mm, all from Bregma). To minimize damage, cells were infused at a controlled rate of 175 nl min^−1^ using a controlled micropump system (World Precision Instruments). Backflow was minimized by leaving the needle in place for an additional 5 min. Experimental animals were compared to HD chimeric littermates that did not receive WT glia and to naïve *Rag1*^*−/−*^ mice that received WT glia at 36 weeks of age following this exact procedure.

#### Human glial striatal isografts

To evaluate the effects of cell age as a determinant of competitive dominance between human glia, newborn *Rag1*^*−/−*^ mice were injected following the same perinatal transplant protocol described above, but instead we delivered glia derived from WT-mCherry to generate human–mouse chimeras harboring WT human glia (WT chimeras). At 40 weeks of age, WT chimeras were then injected following the same adult transplant described above, but instead, we delivered isogenic WT-EGFP glia. Likewise, experimental animals were compared to WT chimeric littermates that did not receive WT-EGFP glia and to naïve *Rag1*^*−/−*^ mice that received WT-EGFP glia at 40 weeks of age following this exact procedure.

Aseptic technique was used for all xenotransplants. All mice were housed in a pathogen-free environment with a 12 h on and off day/night cycle, temperature ranging between 18 °C and 26 °C, humidity between 30% and 70% and ad libitum access to food and water. All procedures were performed in agreement with protocols approved by the University of Rochester Committee on Animal Resources.

### Tissue processing and immunostaining

Experimental animals were perfused with HBSS (Thermo Fisher Scientific, 24020117) followed by 4% PFA. The brains were removed, postfixed for 2 h in 4% PFA and rinsed 3× with PBS. They were then incubated in 30% sucrose solution (Sigma-Aldrich, S9378) until equilibrated, then embedded in OCT in a sagittal orientation (Sakura, 4583), frozen in 2-methylbutane (Thermo Fisher Scientific, 11914421) between −60 °C and −70 °C, and transferred to a −80 °C freezer. The blocks were then cut as 20 µm sections on a CM1950 cryostat (Leica), serially collected on adhesion slides and stored at −20 °C until further use.

Identification and phenotyping of human cells were accomplished by immunostaining for their respective fluorescent reporter, together with a phenotypic marker, including Olig2 (GPCs and oligodendroglia), GFAP (astrocytes) or Ki67 (proliferating cells). Genetically expressed fluorescent reporters were used as markers for human cells, as their expression remained stable throughout the animal’s life (Supplementary Fig. 5). In mice that received a 1:1 mixture of WT-mCherry and WT-untagged human glia, the latter were identified by the expression of human nuclear antigen (hN) and the lack of fluorescent reporter expression.

Antibody sources and concentrations are listed in Supplementary Table 1. Immunolabeled sections were rehydrated with PBS and then incubated in permeabilization/blocking buffer (PBS + 0.1% Triton-X (Sigma-Aldrich, T8787) + 10% normal goat serum (Thermo Fisher Scientific, 16210072)) for 2 h. Sections were then incubated overnight with primary antibodies at 4 °C. The following day, the sections were rinsed with PBS and secondary antibodies were applied for 1 h. After again rinsing with PBS, a second round of primary antibodies, this time against fluorescent reporters, were applied to the sections overnight at 4 °C. These were rinsed with PBS the following day, and the sections were incubated with secondary antibodies for 1 h. The slides were again thoroughly washed with PBS and mounted with Vectashield Vibrance (Vector Labs, H-1800).

### Apoptosis assay

Identification of apoptotic cells within human cell populations was accomplished by TUNEL together with immunostaining for their respective fluorescent reporters. TUNEL was performed using the Click-iT TUNEL Alexa Fluor 647 Imaging Assay (Invitrogen, C10247) following the manufacturer’s instructions with the exception that samples were incubated in Proteinase K solution for 20 min at room temperature. To confirm efficient TUNEL staining in fixed-frozen brain cryosections, positive control sections were treated with DNase I following the manufacturer’s instructions. Following TUNEL, sections were immunolabeled for fluorescent reporters following the previously described immunostaining protocol.

### Quantitative histology

#### Transplant mapping and 3D reconstruction

To map human cell distribution, whole-brain montages of 15 equidistantly spaced, 160 µm apart, sagittal sections spanning the entire striatum were captured using a Nikon Ni-E Eclipse microscope equipped with a DS-Fi3 camera at ×10 magnification and stitched in the NIS-Elements imaging software (Nikon). The striatum within each section was outlined, and immunolabeled human cells were identified and mapped within the outlined striatum using Stereo Investigator (MicroBrightField Bioscience). When applicable, the site of adult injection was mapped as a reference point for volumetric quantification of human cell distribution. Mapped sections were then aligned using the lateral ventricle as a reference to produce a 3D-reconstructed model of the humanized mouse striatum. After 3D reconstruction, the cartesian coordinates for each human cell marker, injection site and striatal outlines were exported for further analysis.

To map the distribution and proportion of mitotically active cells within each human donor cell population, human cells expressing Ki67-immunoreactivity were mapped in every third section of the 15 equidistant sections used to perform the 3D reconstructions. Ki67 quantification was thus done every (160 µm × 3 = ) 480 µm.

#### Volumetric distribution analysis

To quantify the spatial distribution of HD glia in HD chimeras (Extended Data Fig. 1), the volumes of each mapped striatal section were calculated by multiplying the section thickness (20 µm) by the section area. The cell density for each section was then calculated by dividing the number of mapped cells in each section by their respective volume.

To quantify the spatial–temporal dynamics of competing human glia, we developed a program to calculate the volumetric distribution of each cell population as a function of distance to the WT glia delivery site in 3D-reconstructed datasets (Figs. 1 and 3 and Extended Data Figs. 1 and 3). To that end, each quantified section was given an upper and lower boundary ${z}_{u},{z}_{l}$, by representing the striatal outline as two identical polygons separated from each other by the section thickness (20 µm). Then, because the depth-wise location of each cell marker within each individual section is unknown, mapped cells within each section were represented as uniform point probability functions with constant probability across the section. That is, each cell marker in a section from ${z}_{l}$ to ${z}_{u}$ has a probability function as follows:

$$P\left(z\right)=\left\{\begin{array}{c}\frac{1}{{z}_{u}-{z}_{l}},\,{z}_{l}\le z < {z}_{u}\\ 0,\,{\mathrm{otherwise}}\end{array}\right.\,.$$

The spatial distribution of each cell population was then measured by counting the number of mapped cells within concentric spherical shells radiating from the WT glia delivery site in radial increments of 125 µm (for control HD or WT chimeras, an average of the coordinates of the adult WT glia delivery site was used). Mapped cells were counted as 1 if their respective representative line segments were fully inside, 0 if fully outside and partially if intersecting the spherical shell at either the upper or lower boundary of its corresponding section. The density of each cell population ${\rho }_{a,b}$—where *a* and *b* represent the minimum and maximum radii of the spherical shell—was then calculated by dividing the number of mapped cells within the spherical shell by the combined section volume in the shell as follows:

$${\rho }_{a,b}={N}_{a,b}/{V}_{a,b}$$

where ${N}_{a,b}$ is the sum of integrated point probability functions over each section for each point and ${V}_{a,b}$ is the combined section volume within the spherical shell. A detailed description of cell counting and section volume calculation can be found in Supplementary Information. Subsequent analyses were restricted to a 2 mm spherical radius. The code was implemented in Python 3.8 and the package overlap (https://github.com/severinstrobl/overlap) was used to calculate the exact section volume within which the cells were counted.

#### Human cell phenotyping

Quantification of each human cell phenotype (except for Ki67 and TUNEL) was performed using the optical fractionator method^67^ in five equidistant sagittal sections, separated 480 µm apart, spanning the entire striatum. First, whole striatum z-stacked montages were captured using a Nikon Ni-E Eclipse microscope equipped with a DS-Fi3 camera at ×20 magnification and stitched together in NIS-Elements imaging software. Each z-stack tile was captured using a 0.9 µm step size. The montages were then loaded onto Stereo Investigator and outlines of the striatum were defined. A set of 200 × 200 µm counting frames was placed by the software in a systematic random fashion within a 400 × 400 µm grid covering the outlined striatum of each section. Counting was performed in the entire section height (without guard zones), and cells were counted based on their immunolabelling in the optical section in which they first came into focus. Representative images showing whole striata were generated from whole-brain montages using the ‘crop’ function and by adjusting the ‘min/max’ levels in NIS-Elements imaging software.

#### Quantification of TUNEL+ human cells

To assess the distribution and proportion of apoptotic cells within each human cell pool, whole striatal montages of five equidistantly spaced, 480 μm apart, sagittal sections spanning the entire striatum were captured using a Nikon Ni-E Eclipse microscope equipped with a DS-Fi3 camera, at ×10 magnification and stitched in the NIS-Elements imaging software. The striatum was outlined within each section, and immunolabeled human cells were identified and mapped based on their TUNEL labeling within the outlined striatum using Stereo Investigator.

Representative images showing whole humanized striata were generated from previously acquired whole-brain montages using the ‘crop’ function and adjusting the ‘min/max’ levels in NIS-Elements imaging software. Representative images of human glial competitive interfaces were then captured as large field z-stacked montages, using a Nikon Ti-E C2+ confocal microscope equipped with 488 nm, 561 nm and 640 nm laser lines, and a standard PMT detector. Images were captured at ×40 or ×60 magnification with oil-immersion objectives and stitched in NIS-Elements. Maximum intensity projections were then generated, and the ‘min/max’ levels were adjusted in NIS-Elements. Similarly, representative images of human cell phenotype were captured, imaged and processed as z stacks using the Nikon Ti-E C2+ confocal and the same laser lines.

### Fluorescence-activated cell sorting (FACS) of human glia from chimeric mice

To isolate human cells for scRNA-seq, experimental chimeras were perfused intracardially with HBSS, their striata dissected and tissue dissociated as previously described^68^, and as illustrated in Supplementary Fig. 7a. Briefly, mice were killed with euthasol, transcardially perfused with sterile HBSS containing magnesium chloride and calcium chloride and their brains removed. The brains were immersed in ice-cold sterile HBSS for about 5 min to facilitate the microdissection. Under a dissecting microscope, the striata from each mouse was dissected and placed in sterile HBSS on ice. The striatal tissues were transferred to a Petri dish containing sterile HBSS without magnesium chloride and calcium chloride, chopped into small pieces using sterile disposable scalpels, transferred into a sterile tube and then incubated in a papain/DNase dissociation solution at 37 °C for 50 min. Ovomucoid dissolved in EBSS was then added to inactivate the papain. The tissue was triturated by repeated pipetting to achieve a single-cell suspension. The cells were then pelleted, resuspended into MEM and filtered for flow cytometry. Single-cell preparations were isolated based on their expression of mCherry, EGFP, or their absence, using a BD FACSAria Fusion (BD Biosciences, FACS Diva Software). To exclude dead cells, 4′,6-diamidino-2-phenylindole (Thermo Fisher Scientific, D1306) was added at 1 µg ml^−1^. Our gating strategy is shown in Supplementary Fig. 7b.

### scRNA-seq analysis

#### Primary data acquisition

Isolated cells were captured for scRNA-seq on a 10X Genomics chromium controller (v3.1 chemistry). Libraries were generated according to the manufacturer’s instructions and sequenced on an Illumina NovaSeq 6000 (NovaSeq Control Software) at the University of Rochester Genomics Center. scRNA-seq libraries were aligned with STARsolo^69,70^ using a custom two-pass strategy. First, an annotated chimeric GRCh38 and GRCm38 reference were generated using Ensembl 102 human and mouse annotations, with the addition of mCherry and EGFP. STARsolo was then run with the following parameters: twopassMode=basic, limitSjdbInsertNsj=3000000, and soloUMIfiltering= MultiGeneUMI. BAM files were then split by species, and cross-species multimapping reads were assigned to both human or mouse BAMs. FASTQ files were regenerated from either the mouse or human BAM files and re-aligned to a single species reference. STARsolo was run again with the following parameters: twopassMode=basic, limitSjdbInsertNsj=2000000, and soloUMIfiltering= MultiGeneUMI.

#### Differential expression analysis

Human data were imported into R^71^ using Seurat^72^. Cells were filtered (unique genes >250 and percent mitochondrial genes <15). Cells were then further filtered for expression of mCherry or EGFP. Counts were imported into Python for integration using scvi, where the 4,000 most variable features were used^73^. The model was trained for integration using the mouse sample and cell line in addition to the number of unique genes and percent mitochondrial gene expression. The latent representation was then used for dimensionality reduction via uniform manifold approximation projection (UMAP) and Louvain community detection. Smaller populations of cells were classified into six major types of glia based on marker expression. Data were then re-imported into Seurat, and differential expression was carried out using model-based analysis of single-cell transcriptomics (MAST)^74^. Genes were considered for differential expression if their expression was detected in at least 3% of all GPCs. The model design for differential expression used the number of unique genes in a cell and the experimental group (cell line/age of the cell, and if the cell was in the presence or absence of an opposing clone). Significance for differential expression was *P* < 0.05, with a log_2_ fold change of at least 0.15. IPA (QIAGEN) was used for functional analysis of each differentially expressed gene list.

#### Cell cycle analysis

G2M scores of each experimental group were calculated using Seurat’s CellCycleScoring function. Statistical comparisons between each model’s experimental groups were then calculated using Dunn tests with Benjamini–Hochberg multiple comparison adjustments.

#### Identification of TF-associated regulons

Genes were first filtered to retain only those that expressed at least three counts in at least 1% of the cells. We used all 10,410 cells in this analysis. The filtered raw matrix was then used as input for the standard pipeline of pySCENIC^75^ to identify each TF and its putative downstream targets in our dataset. These gene sets are referred to as regulons and are assigned ‘area under the curve’ (AUC) values to represent their activities in each cell, with higher values indicating a stronger enrichment of such regulon. We then used the resulting AUC matrix to look for important TFs. Within the GPC subpopulation in both our isograft and allograft models, we assigned 1 to cells from the young WT samples and 0 to cells from the aged WT or aged HD samples. Lasso logistic regression was then performed on predetermined 0/1 outcome with all TF’s AUCs as predictors using glmnet. Lambda for logistic regression was automatically defined with cv.glmnet.

We isolated TFs with positive coefficients and further filtered based on their mean activity per group, such that TF mean activity in the young WT should be higher than that in the aged counterpart. The final step was to perform gene set enrichment analysis^76^ on regulons identified thus far, to determine if they were enriched for differentially upregulated genes in young WT cells compared to aged HD and WT cells (adjusted *P* < ×10^−3^, NES > 0).

#### Identification of co-expressed gene sets with competitive advantage

We filtered to exclude genes with fewer than 1 count across all cells and used the resulting matrix to denoise data with DCA^77^. WGCNA^35^ was performed on denoised data of the GPC subset. A signed network adjacency was calculated with soft thresholding power of 19. Modules were detected after hierarchical clustering of genes on topological overlap matrix-based dissimilarity and dynamic tree cut. We then identified modules whose gene members represented a significant overlap with the important TF targets identified above, using GeneOverlap (adjusted *P* < 10^−2^)^78^. The relative contribution of the linearly independent covariates age (young, aged) and genotype (HD, WT) toward the additive explanation for each module eigengene (for example, ME ~ age + genotype) was calculated by the lmg method, implemented in the relaimpo package^79^.

#### Network representation

Functional annotation of TFs’ gene targets was performed with IPA^80^. To create a representative network, we focused on the MYC regulon and its shared targets with other important TFs. Networks were constructed with Cytoscape^81^.

### Statistical analysis and reproducibility

Samples exhibiting artifacts related to technical issues from experimental procedures—such as mistargeted injections, or overt surgical damage—were excluded from this study (see Supplementary Table 2 for a complete list of injected mice). Statistical tests were performed using GraphPad Prism 9, and all tests used are indicated in each figure caption. For comparisons between more than two groups, one-way analysis of variance with Tukey’s or Šidák’s tests for multiple comparisons were applied. For comparisons between two groups with more than two factors, two-way analysis of variance with Šidák’s multiple comparison test was applied. When comparing two unmatched groups, unpaired two-tailed *t* tests were applied. Significance was defined as *P* < 0.05. Respective *P* values are stated in the figures whenever possible; otherwise, ****P < 0.0001, ***P < 0.001, **P < 0.01 and *P < 0.05. The number of replicates is indicated in the figure legends, with *n* denoting the number of independent experiments. Data are represented as the mean ± s.e.m.

### Resource and reagent availability

Further information and requests for resources and reagents should be directed to and will be fulfilled by the senior author S.A.G.

### Reporting summary

Further information on research design is available in the Nature Portfolio Reporting Summary linked to this article.

## Online content

Any methods, additional references, Nature Portfolio reporting summaries, source data, extended data, supplementary information, acknowledgements, peer review information; details of author contributions and competing interests; and statements of data and code availability are available at 10.1038/s41587-023-01798-5.

### Supplementary information

Supplementary Information Supplementary Methods, Supplementary Figs. 1–7 and Supplementary Tables 1 and 2.

Reporting Summary

Supplementary Table 3 Differentially expressed gene lists in the HD allograft model.

Supplementary Table 4 IPA terms from the HD allograft model.

Supplementary Table 5 Differentially expressed gene lists in the isograft model.

Supplementary Table 6 IPA terms from the isograft model.

Supplementary Data 1 Source data for Supplementary Fig. 3.

Supplementary Data 2 Source data for Supplementary Fig. 5.

### Source data

Source Data Fig. 1 Statistical source data.

Source Data Fig. 2 Statistical source data.

Source Data Fig. 3 Statistical source data.

Source Data Fig. 4 Statistical source data.

Source Data Fig. 5 Statistical source data.

Source Data Extended Data Fig. 1 Statistical source data.

Source Data Extended Data Fig. 2 Statistical source data.

Source Data Extended Data Fig. 3 Statistical source data.

Source Data Extended Data Fig. 4 Statistical source data.

Source Data Extended Data Fig. 5 Statistical source data.

Source Data Extended Data Fig. 6 Statistical source data.

## Data Availability

The sequencing datasets reported in this paper can be accessed at GEO, via accession number GSE206322. Source data are provided with this paper.

## Code availability

Our program for the quantification of mapped cell populations in 3D-reconstructed tissues is publicly available through the following link: QIM/Tools/Thick Section Point Density GitLab (dtu.dk).

All codes used to analyze and generate figures for the genomics dataset are accessible on GitHub at: https://github.com/CTNGoldmanLab/HD_Competition_2022
