# Supplementary material for: Young glial progenitor cells competitively replace aged and diseased human glia in the adult chimeric mouse brain
Source: Nat Biotechnol. 2023 Jul 17;42(5):719–30. doi: 10.1038/s41587-023-01798-5 (PMC11098747; doi:10.1038/s41587-023-01798-5)
Supplement: Supplementary file 1 — Supplementary Methods, Supplementary Figs. 1–7 and Supplementary Tables 1 and 2. [file 41587_2023_1798_MOESM1_ESM.pdf]

nature biotechnology

Article https://doi.org/10.1038/s41587-023-01798-5

Young glial progenitor cells competitively replace aged and diseased human glia in the adult chimeric mouse brain

In the format provided by the authors and unedited

Young glial progenitor cells competitively replace aged and diseased human glia in the adult chimeric mouse brain

Vieira et al.

SUPPLEMENTARY INFORMATION

Included:

Supplementary Methods

7 Supplementary Figures

2 Supplementary Tables

Supplementary Methods

Embryoid Body (EB) generation

To generate uniform EBs, hESCs were dissociated to small clusters with ReLeSR, harvested, and counted with the automated cell counter NucleoCounter NC-200. A total of 3 × 10^6 hESCs were added per well of a AggreWell-800 plate (StemCell Technologies, cat. no. 34815) and centrifuged to aggregate the hESCs in the individual microwells. Aggregated hESCs were cultured overnight with mTeSR1 supplemented with 10 µm Y-27632 to allow for EB formation. 24h following aggregation, EBs were released from each microwell by gently pipetting medium in each well using a P1000 pipette with a cut tip and transferred into ultra-low attachment tissue culture flasks (Corning, cat. no. 3815) for further directed differentiation. Prior to aggregation, the AggreWell-800 plates were prepared according to the manufacturer's guideline.

Flow cytometry of hESC-derived glial cultures

Glial cultures were collected as a single cell suspension following 5 min dissociation in Accutase, counted with a hemocytometer, and resuspended at 10^6 cell/ml in Miltenyi wash buffer (MWB; PBS + 0.5% BSA Fraction V (ThermoFisher cat. no. 15260037) + 2 µM EDTA (ThermoFisher cat. no. 15575020)). Each cell suspension was then incubated in MWB for 15 mins at 4ºC to block non-specific antibody binding and divided in 100 µL fractions for immunolabelling. Each fraction was then incubated with fluorophore-conjugated antibodies for 15 min at 4°C, except for the unstained gating controls. Antibody sources and concentrations are listed in **Supplementary Table 1**. Cells were then washed with MWB, spun for 10 min at 200 x g, resuspended in MWB, and strained into 5 ml polystyrene tubes with 35 µm cell-strainer caps (Corning, cat. no. 352235). To exclude dead cells, 4′,6-diamidino-2-phenylindole (DAPI; ThermoFisher cat. no. D1306) was added at 1 µg/mL. Flow cytometry analysis of glial cultures was then performed on a CytoFLEX S platform (Beckman Coulter), and the data analysed with the CytExpert (Beckman Coulter) and FlowJo (BD Biosciences) software. Gating strategy and data analysis are exemplified in **Supplementary Fig. 4**.

Immunocytochemistry of hESC and glial cultures

Cultures were fixed with 4% paraformaldehyde (PFA) for 7 mins, washed with phosphate-buffered saline (PBS) and then permeabilized and blocked with permeabilization/block buffer (PBS + 0.1% Triton-X (Sigma-Aldrich cat. no. T8787) + 1% BSA Fraction V) for 1h. Cultures were then incubated overnight with primary antibodies at 4ºC, washed with PBS, and then incubated with secondary antibodies at room temperature for 2h. Antibody sources and concentrations are listed in **Supplementary Table 1**. Nuclear counterstain was then performed by incubating with 1 µg/mL DAPI for 5 mins at room temperature, and then washed with PBS an additional 3 times prior to imaging.

Representative images of hESCs were acquired on a Nikon Eclipse Ti microscope equipped with a DS-Fi3 camera at 10x magnification while representative images of glial cultures were captured with a DS-Qi2 camera at 20x magnification, and 'min/max' levels were adjusted for both in NIS-Elements imaging software (Nikon).

Mapped cell counting and section volume estimation (Volumetric Quantification)

As previously mentioned in the methods section, mapped cells are counted as 1 if their respective representative line segments are fully inside, 0 if they are fully outside, and partially if they are intersecting the radiating spherical shell. To that end, we calculate the points on the surface of the radiating spherical shells corresponding to the projection of mapped cells onto the spherical shell. We here consider only the calculation of points above the injection site since points below are similarly handled. The corresponding points on the spherical shell are given by:

$$z_d(r) = \sqrt{r^2 - d_{xy}^2}$$

where r is either *a* or *b*, depending on the if the shell intersects the point at the outer or inner surface. If $z_l > z_d(b)$, the point is outside the shell and thus not counted, if $z_u < z_d(a)$, the shell has passed beyond the point and it is not counted. If $z_d(a) < z_u$ and $z_d(b) > z_l$, the line segment is completely within the spherical shell and it is counted as 1. Additionally, we may have the two limiting cases where the spherical shell intersects the line segment. These two examples are similar, so we deal only with the case where the line segment intersects the upper surface of the spherical shell. That is, the case where $z_d(a) > z_u$ and $z_u < z_d(b) < z_l$. In this case, the part of the line segment inside the spherical shell has length $z_u - z_d(b)$ and we count the mapped cell by integrating its point probability function as:

$$(z_u - z_d(b))/_{w}$$

where *w* is the width of the section.

To calculate the corresponding section volume within which mapped cells were counted, we first triangulate each polygon representing the anatomical boundary of each section. We then form a prism from the triangles with height matching the section thickness. We represent each prism as

3 tetrahedra and measure to cumulative volume inside the sphere as the total overlap volume between the sphere of radius r and each tetrahedra.

For a section with depth coordinate z_l, each triangle is represented by 3 points v_1, v_2, v_3 with coordinates (x_1, y_1, z_l), (x_2, y_2, z_l), and (x_3, y_3, z_l). These triangles together make a 2D representation of the domain. To get a 3D representation of each section, we use the known thickness of dz (here 20 nm) and thicken the triangle into a prism shape with 3 new points w_1, w_2, w_3 translated perpendicular the section plane by dz upon the upper boundary of the section at coordinates (x_1, y_1, z_u), (x_2, y_2, z_u), and (x_3, y_3, z_u). Calculating the exact overlap volume of a 3D polygon and a sphere is not trivial, but we can calculate the overlap volume of spheres and tetrahedra. To that end, we cover each prism domain by 3 tetrahedra given by the coordinate sets $\langle v_1, v_2, v_3, w_1 \rangle$, $\langle v_2, v_3, w_1, w_2 \rangle$, and $\langle v_3, w_1, w_2, w_3 \rangle$. Given a sphere of radius r, the intersecting volume of each section with the sphere is then given by the sum over the volume of the intersection of a sphere *S* of radius *r* and each tetrahedra *T*:

$$V(r) = \sum_T |S_r \cap T|$$

Supplementary Figures

Supplementary Figure 1

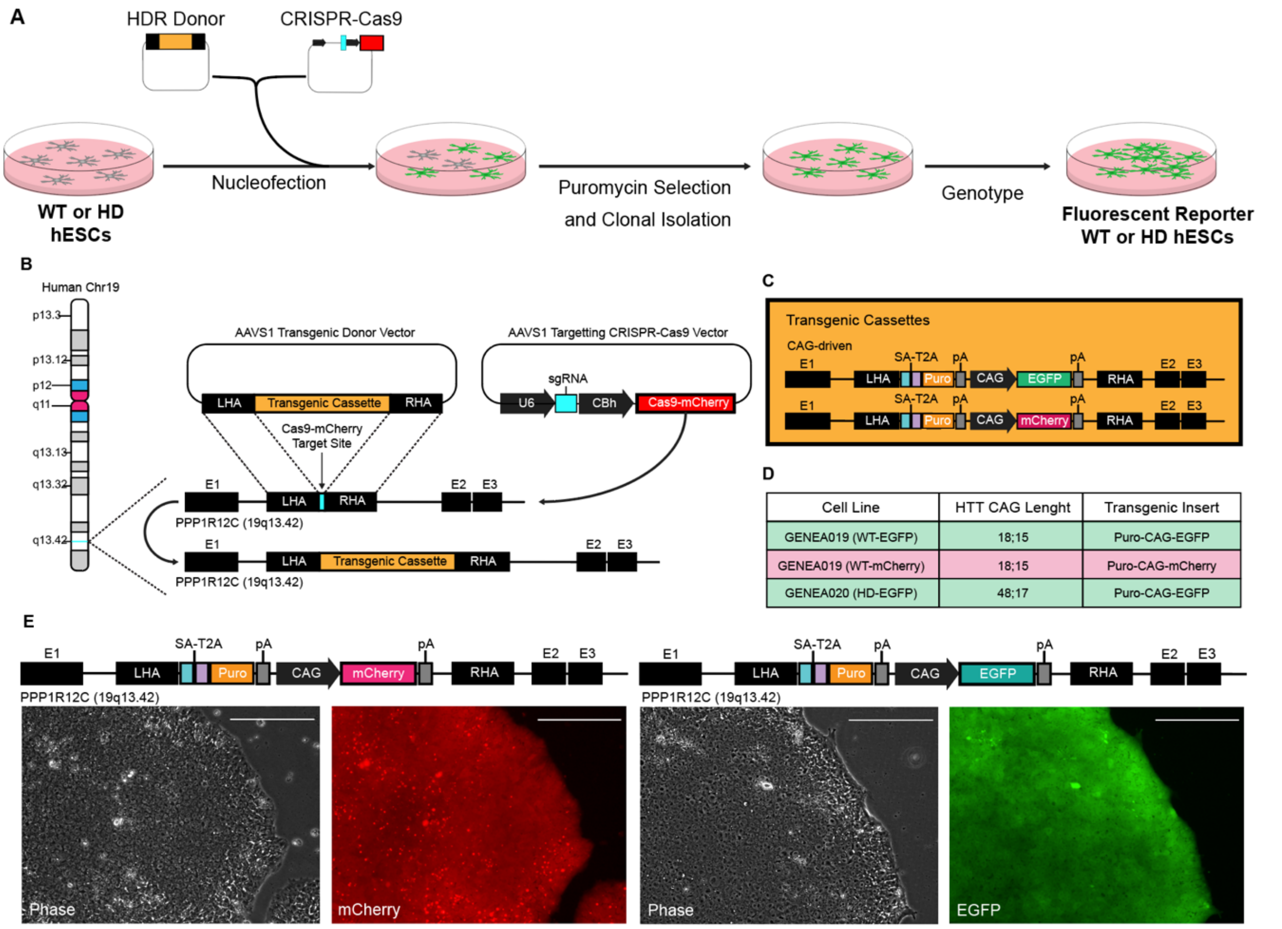

Generation of fluorescent reporter WT and HD hESCs

A. Workflow employed in the genetic engineering of the adeno-associated virus integration site 1 (AAVS1) locus of hESC lines to constitutively express transgenes of interest. **B.** Mechanism of CRISPR-Cas9 mediated transgene integration into the AAVS1 locus (located in the first intron of the protein phosphatase 1 regulatory subunit 12C (PPP1R12C) gene). **C.** Table of the different transgenic cassettes integrated into the AAVS1 locus of hESCs. Donor cassettes use a gene-trap selection approach[2] where puromycin resistance is driven from the endogenous AAVS1 promoter to enhance clonal selection. **D**. Table describing the engineered WT and HD hESC lines' HTT CAG length and respective transgenic insert. **E**. CRISPR-mediated integration of transgenic reporter cassette into the AAVS1 safe harbour locus yields color-tagged WT and HD hESCs, that express mCherry (*red*) or EGFP (*green*). **B-C.** E1-3, exon 1-3; LHA, left homology arm; SA, splice acceptor site; T2A, 2A self-cleaving peptide; Puro, Puromycin resistance gene; pA, polyadenylation sequence; CAG, CAG promoter; RHA, right homology arm. Scale: **E**, 500 µm.

Supplementary Figure 2

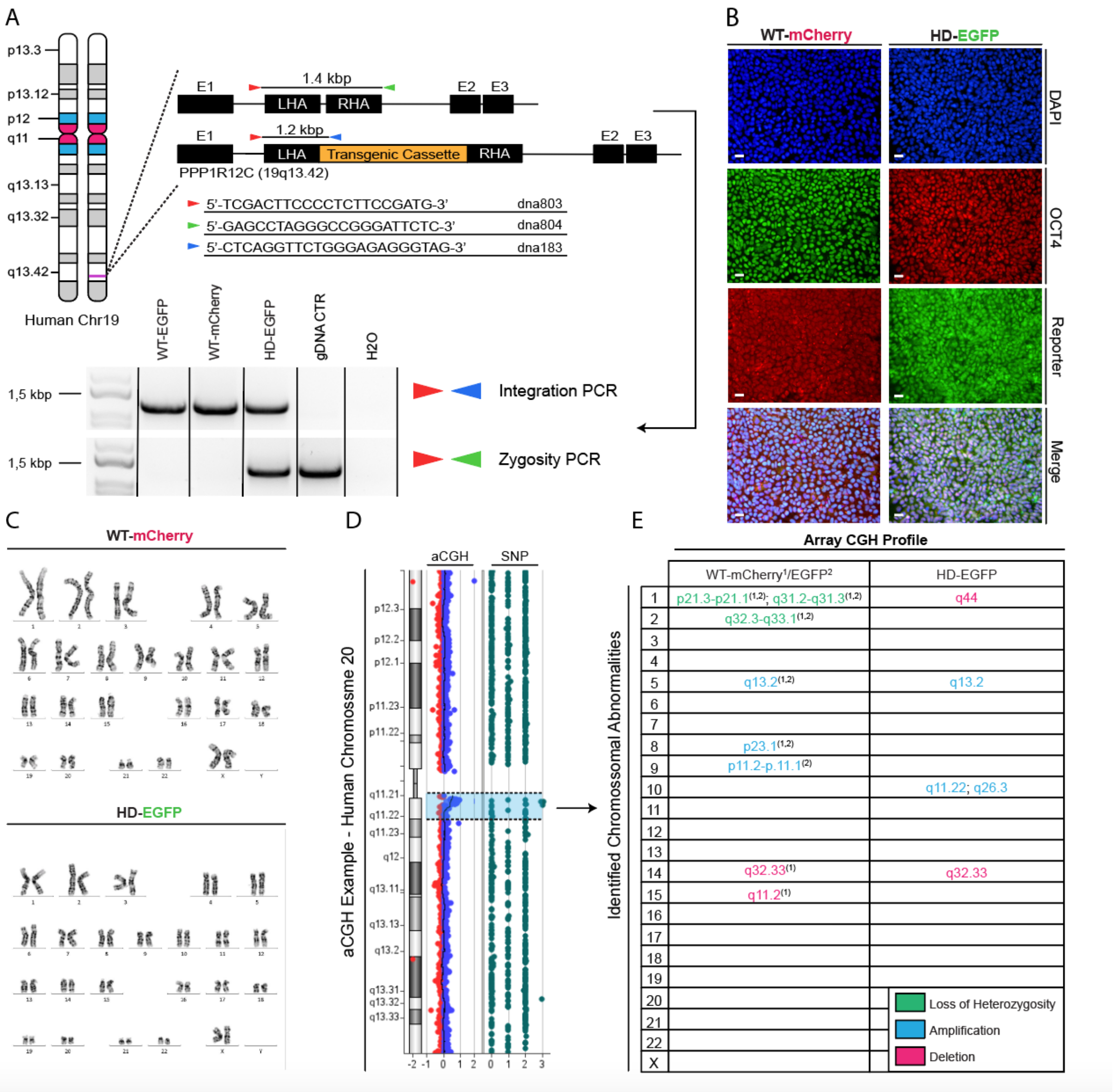

	WT-mCherry[1]/EGFP[2]	HD-EGFP
1	p21.3-p21.1[(1,2)]; q31.2-q31.3[(1,2)]	q44
2	q32.3-q33.1[(1,2)]	
3		
4		
5	q13.2[(1,2)]	q13.2
6		
7		
8	p23.1[(1,2)]	
9	p11.2-p.11.1[(2)]	
10		q11.22; q26.3
11		
12		
13		
14	q32.33[(1)]	q32.33
15	q11.2[(1)]	
16		
17		
18		
19		
20		Loss of Heterozygosity
21		Amplification
22		Deletion
X		

Characterization of fluorescent reporter WT and HD hESCs

A. PCR screening strategy to assess transgene cassette integration and zygosity. PCR screening shows that WT-EGFP, WT-mCherry and HD-EGFP integrated the transgenic cassette in the correct site, with WT-mCherry and WT-EGFP harboring a homozygous integration while HD-EGFP harbors a heterozygous integration. E1-3, exon 1-3; LHA, left homology arm; RHA, right homology arm. **B**. Immunostaining for OCT4 shows that pluripotency is maintained following transgene insert. **C.** Karyotyping shows that no chromosomal abnormalities were acquired during the transgene integration process. **D.** Example of an aCGH profile of human chromosome 20 carrying an amplification commonly found in hESCs (within the dashed lines), known to impart a selective growth advantage. No such mutation was detected in WT-EGFP, WT-mCherry or HD-EGFP hESCs. **E**. Comparative aCGH profiling detected multiple mutations in the engineered lines, within and outside of normal range. None were predicted to influence experimental outcomes.

Supplementary Figure 3

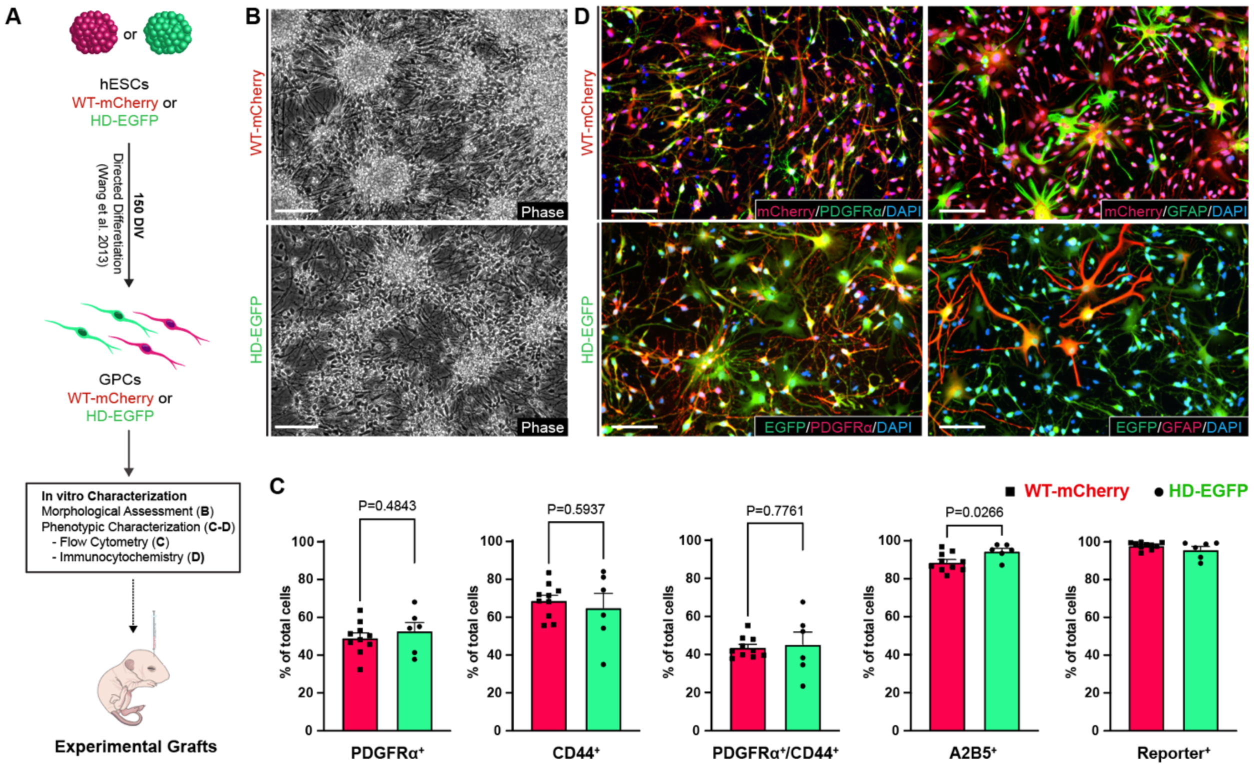

Derivation of hGPCs from reporter WT and HD hESCs

A. Schematic illustrating the differentiation process and phenotypic characterization prior to experimental grafting. **B**. Phase-contrast images of WT-mCherry and HD-EGFP glial cultures, both highly enriched in bipolar hGPCs at 150 DIV. Images are representative of n=10 WT-mCherry, and n=6 HD-EGFP glial cultures. **C.** Flow cytometry of 150 DIV cell preparations (WT-mCherry, n=10; HD-EGFP, n=6) reveals high enrichment of CD140a (PDGFRα)$^{+}$/CD44^{+} hGPCs, with the remainder comprised of less mature A2B5^{+} hGPCs and PDGFRα^{-}/CD44^{+} astrocytes. Fluorescent reporter expression remained consistent throughout glial differentiation. **D.** Immunocytochemistry confirmed the enrichment of PDGFRα^{+} hGPCs in cultures generated from both WT-mCherry and HD-EGFP hESCs. A fraction of these hGPCs differentiated into GFAP^{+} astrocytes. Images are representative of n=6 WT-mCherry, and n=7 HD-EGFP glial cultures. **C**, Unpaired two-tailed t tests; data are shown as means ± SEM. Scale: **B,** 50 μm; **D**, 100 μm.

Supplementary Figure 4

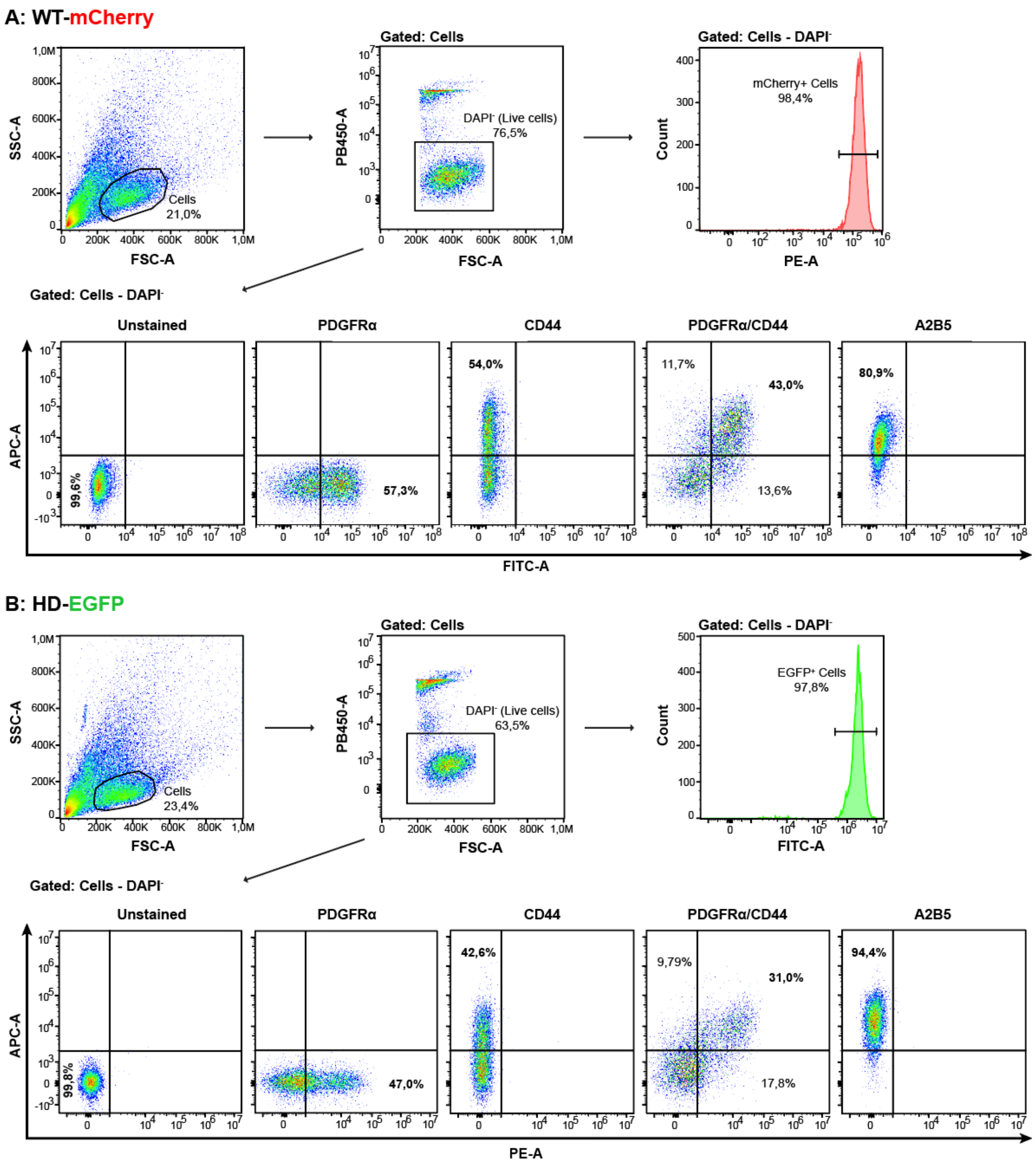

Gating strategy for flow cytometry characterization of glial cultures

A-B. Examples of the gating strategy employed in the flow cytometry characterization of glial cultures derived from both reporter WT-mCherry (**A**) and HD-EGFP (**B**) hESC lines. From dissociated glial cultures, live cells were identified by their lack of DAPI incorporation. Of these, cells stained for PDGFRα, CD44, PDGFRα/CD44 and A2B5 were identified based on antibody-specific fluorescence intensity, relative to their respective unstained gating controls. Essentially all cells retained their respective reporter expression throughout glial differentiation in vitro.

Supplementary Figure 5

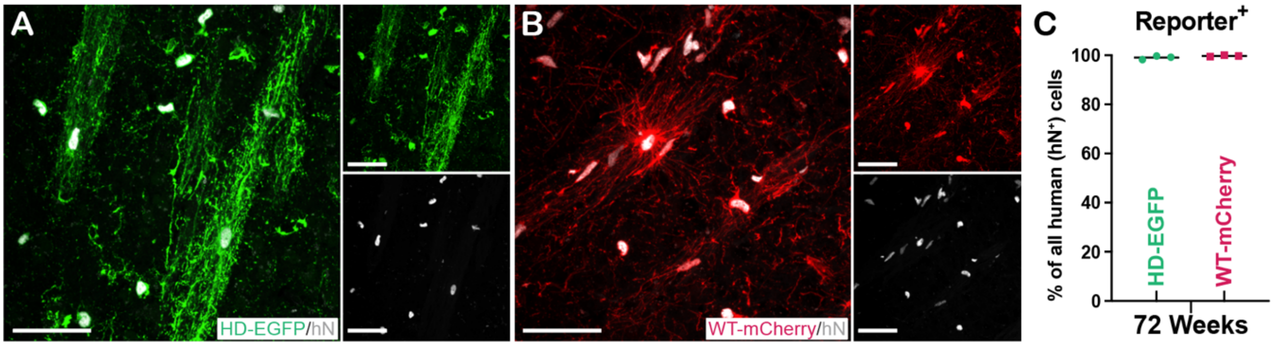

Reporter expression is uniformly maintained throughout long-term *in vivo* maturation

A-C – Immunolabeling for human nuclear antigen (hN^{+}, white) in 72 week-old mice engrafted with either HD-EGFP glia (**A**, n=3) or WT-mCherry glia (**B**, n=3). **C.** Expression of both fluorescent reporters remained consistent and ubiquitous during expansion and subsequent differentiation of transgenic human glia, in vivo, with essentially all human $nuclei^{+}$ cells expressing their tagged fluorescent transgene at 72 weeks in vivo. n=3 for each experimental group. Scale: **A**-**B**, 50 µm.

Supplementary Figure 6

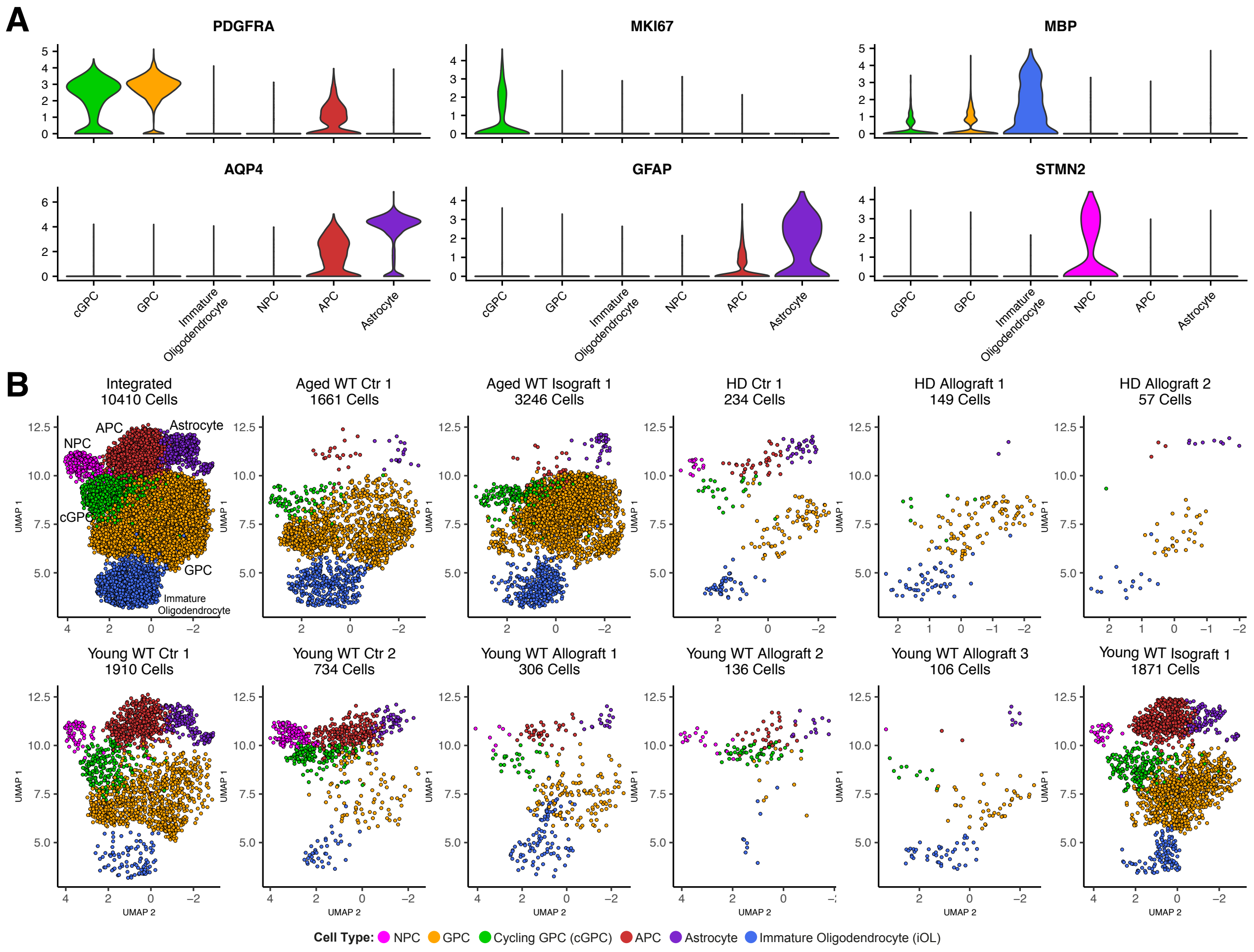

scRNA-seq capture characterization

A. Violin plots of cell type marker expression across all groups.

B. UMAP plots of biological replicate captures and corresponding human cell numbers. Top row: Aged, neonatally-transplanted cells in both allograft and isograft paradigms. Bottom row: Younger, later-transplanted cells in both the allograft and isograft paradigms.

Notes on samples run for single cell capture:

For the allograft paradigm (Young WT into Older HD) the following groups were used (1-3 mice pooled/group): Young WT Ctr, Young WT Allograft, HD Ctr, and HD Allograft. Lower yields of striatal HD cells in the allograft paradigm were noted as these were the cells that were largely replaced by the later-transplanted WT cells. (Of note, HD allograft 3 was lost technically due to a reagent failure, so there is no match to the young WT allograft 3.)

For the isograft paradigm (Young WT into Older WT, isogenic) the following groups were used: Young WT Ctr, Young WT Isograft, Aged WT Ctr, and Aged WT Isograft (1-3 mice/group).

Supplementary Figure 7

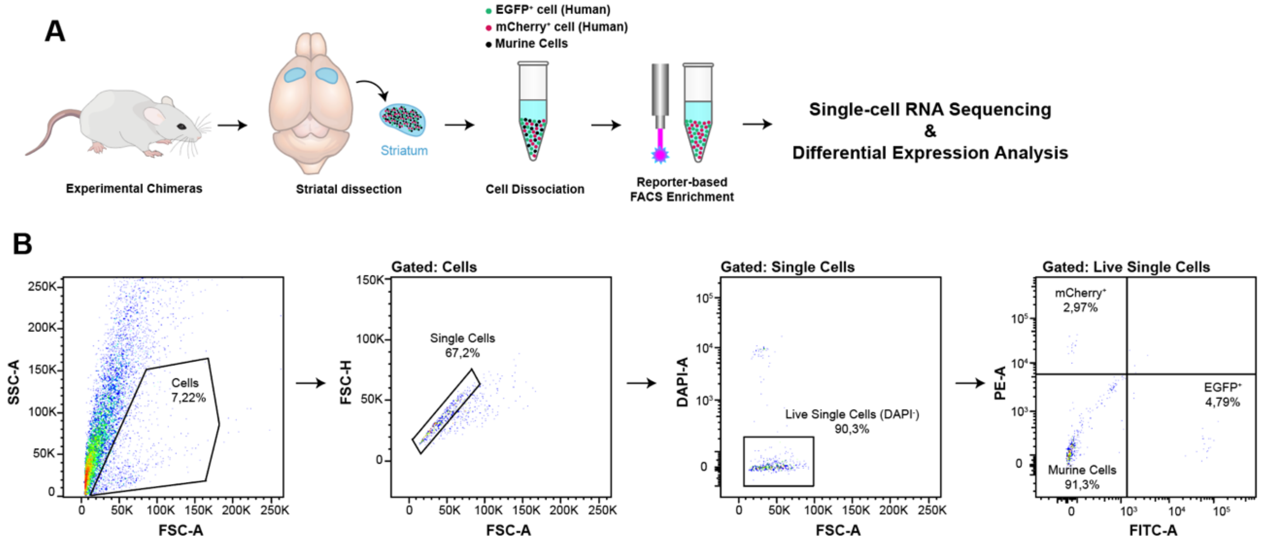

Isolation of implanted human cells from their chimeric hosts

A. Schematic illustrating the experimental workflow involved in the isolation of human cells from the striata of their chimeric hosts. **B**. Example of the gating strategy employed in the FACS enrichment of human cells extracted from dissociated chimeric striata. Live cells were identified by their lack of DAPI incorporation. Of these, human cells were sorted based on their expression of their respective fluorescent reporter ($EGFP^+$ or $mCherry^+$), and harvested for single-cell sequencing and downstream analysis.

Supplementary Tables

Supplementary Table 1
Antibody sources and dilutions

Type	Antigen	Host Species	Dilution	Manufacturer	Catalog Number
Primary	Olig2	Mouse	1:200	Millipore	MABN50
Primary	hGFAP	Mouse	1:200	Biolegend	SMI-21
Primary	hN	Mouse	1:200	Abcam	ab254080
Primary	Ki67	Rabbit	1:200	Invitrogen	MA5-14520
Primary	EGFP	Chicken	1:500	Invitrogen	A10262
Primary	mCherry	Rat	1:500	Invitrogen	M11217
Primary	PDGFRa	Rabbit	1:200	Cell Signalling	5241S
Primary	Oct-4	Mouse	1:100	Millipore	MAB4401
Secondary	Rat IgG (H+L) - Alexa Flour 568	Goat	1:400	Invitrogen	A-11077
Secondary	Chicken IgY (H+L) - Alexa Flour Plus 488	Goat	1:400	Invitrogen	A32931
Secondary	Rabbit IgG (H+L) - Alexa Fluor Plus 647	Goat	1:400	Invitrogen	A32733
Secondary	Mouse IgG (H+L) - Alexa Fluor Plus 647	Goat	1:400	Invitrogen	A32728
Conjugated	CD140a-FITC	Mouse	1:10	BD Horizon	564594
Conjugated	CD140a-PE	Mouse	1:10	BD Pharmingen	556002
Conjugated	CD44-APC	Mouse	1:500	Miltenyi Biotec	130-113-331
Conjugated	A2B5-APC	Mouse	1:50	Miltenyi Biotec	130-093-582

This table lists all antibodies and their respective concentrations used in the histological analysis of experimental chimeras, as well as in all *in vitro* characterization prior to transplant.

Supplementary Table 2

All mice injected in this study

**Sample exclusion criteria and assessment: HD vs WT Group**	**n=25**
Healthy adult graft site with cell expansion	15
Neonatal injection failure (Absence of HD glia)	0
Mistargeted neonatal injection	0
Adult injection failure (Absence of WT cells)	0
Mis-targeted adult injection	6
Overt surgical damage	4

**Sample exclusion criteria and assessment: HD Control Group**	**n=22**
Healthy neonatal graft site with cell expansion	18
Neonatal injection failure (Absence or HD glia)	2
Mistargeted neonatal injection	1
Overt surgical damage	1

**Sample exclusion criteria and assessment: WT Control Group**	**n=15**
Healthy adult graft site with cell expansion	12
Adult injection failure (Absence of WT cells)	2
Mis-targeted adult injection	0
Overt surgical damage	1

**Sample exclusion criteria and assessment: Aged vs Young Group**	**n=14**
Healthy adult graft site with cell expansion	7
Neonatal injection failure (Absence or WT glia)	0
Mis-targeted neonatal injection	0
Adult injection failure (Absence of WT cells)	3
Mistargeted adult injection	2
Overt surgical damage	2

**Sample exclusion criteria and assessment: Aged Control Group**	**n=10**
Healthy adult graft site with cell expansion	9
Neonatal injection failure (Absence or HD glia)	1
Mis-targeted neonatal injection	0
Overt surgical damage	0

**Sample exclusion criteria and assessment: Young Control Group**	**n=14**
Healthy adult graft site with cell expansion	6
Adult injection failure (Absence of WT cells)	8
Mis-targeted adult injection	0
Overt surgical damage	0
