# Supplementary material for: Young glial progenitor cells competitively replace aged and diseased human glia in the adult chimeric mouse brain
Source: Nat Biotechnol. 2023 Jul 17;42(5):719–30. doi: 10.1038/s41587-023-01798-5 (PMC11098747; doi:10.1038/s41587-023-01798-5)
Supplement: Supplementary file 2 — Reporting Summary [file 41587_2023_1798_MOESM2_ESM.pdf]

nature portfolio

Corresponding author(s): Goldman, SA

Last updated by author(s): Apr 3, 2023

Reporting Summary

Nature Portfolio wishes to improve the reproducibility of the work that we publish. This form provides structure for consistency and transparency in reporting. For further information on Nature Portfolio policies, see our Editorial Policies and the Editorial Policy Checklist.

Statistics

For all statistical analyses, confirm that the following items are present in the figure legend, table legend, main text, or Methods section.

n/a	Confirmed	
☐	☒	The exact sample size (n) for each experimental group/condition, given as a discrete number and unit of measurement
☒	☐	A statement on whether measurements were taken from distinct samples or whether the same sample was measured repeatedly
☐	☒	The statistical test(s) used AND whether they are one- or two-sided<br>*Only common tests should be described solely by name; describe more complex techniques in the Methods section.*
☒	☐	A description of all covariates tested
☐	☒	A description of any assumptions or corrections, such as tests of normality and adjustment for multiple comparisons
☐	☒	A full description of the statistical parameters including central tendency (e.g. means) or other basic estimates (e.g. regression coefficient) AND variation (e.g. standard deviation) or associated estimates of uncertainty (e.g. confidence intervals)
☐	☒	For null hypothesis testing, the test statistic (e.g. F, t, r) with confidence intervals, effect sizes, degrees of freedom and P value noted<br>*Give P values as exact values whenever suitable.*
☒	☐	For Bayesian analysis, information on the choice of priors and Markov chain Monte Carlo settings
☒	☐	For hierarchical and complex designs, identification of the appropriate level for tests and full reporting of outcomes
☒	☐	Estimates of effect sizes (e.g. Cohen's d, Pearson's r), indicating how they were calculated

Our web collection on statistics for biologists contains articles on many of the points above.

Software and code

Policy information about availability of computer code

Data collection	CytExpert v.2.0.4.28 was used to acquire in vitro flow cytometry data.<br>BD FACS Diva v.8.0.3 was used for cell sorting from experimental chimeras.<br>NIS-Elements v4.5.0, v4.6.0 and v.5.2.0 was used to acquire and prepare microscopy data.<br>NovaSeq Control Software v.1.6 was used for sequencing.
Data analysis	FlowJo v.10.8 was used to analyze and plot flow cytometry data.<br>Stereo Investigator v.2019 was used to perform quantitative histology.<br>Graph Pad Prism v.9 was used to perform statistical analysis on flow cytometry and quantitative histology data.<br>Scripts used to analyze volumetric cell distribution are available at the following repository upon publication: https://lab.compute.dtu.dk/QIM/tools/thick-section-point-density<br><br>For the analysis of single-cell RNA sequencing data, we used anaconda (v4.4.0 and v4.5.1) for version control, and employed the following software in our workflow:<br>Data Preprocessing: STAR (v2.7.7a), samtools (v1.11)<br>Data analysis was performed in:<br>R (v4.1.1) with the following key libraries: fgsea (v1.20.0), GeneOverlap (v1.30.0), ggplot2 (v3.3.5), relaimpo (v2.2-6), glmnet (v.4.1-4), jsonlite (v1.7.3), Seurat (v4.1.1), SeuratObject (v4.1.0), dplyr (v1.0.8), Dplyr (v 1.0.2), Magrittr (v1.5), Stringr (v1.4.0), Hmisc (v4.3-0), Data.table (v.1.12.6), Tidyr (v1.0.0), ggVennDiagram (v.0.3), Plyr (v1.8.6), DT (v0.11), Patchwork (v.1.0.0),<br>R (v3.5.1) with the following key libraries: WGCNA (v.169)<br>python (v3.7.9) with the following key packages: pySCENIC (v0.11.0), aboreto (v0.1.6), pickleshare (v0.7.5), pandas (v1.2.2), numpy (v1.17.0),

dask (v2021.2.0)
python (v3.8.12) with the following key packages: scanpy (v1.4.4.post1), pandas (v1.1.5), anndata (v0.6.22.post1), dca (v0.3.4), scikit-learn (v1.0.2), matplotlib (v3.5.1), seaborn (v0.11.2), numpy (v1.21.5), scVI (v.0.6.8)
Ingenuity Pathway Analysis v. 01-20-04
Scripts used to analyze scRNAseq data are available at the following repository upon publication: https://github.com/CTNGoldmanLab/HD_Competition_2022

For manuscripts utilizing custom algorithms or software that are central to the research but not yet described in published literature, software must be made available to editors and reviewers. We strongly encourage code deposition in a community repository (e.g. GitHub). See the Nature Portfolio guidelines for submitting code & software for further information.

Data

Policy information about availability of data

All manuscripts must include a data availability statement. This statement should provide the following information, where applicable:

- Accession codes, unique identifiers, or web links for publicly available datasets
- A description of any restrictions on data availability
- For clinical datasets or third party data, please ensure that the statement adheres to our policy

Source data for the derivation and characterization of reporter hGPCs and quantitative histology is provided herein.
scRNA sequencing data were deposited to GEO and available at GSE206322.
scRNA-sequencing data were aligned to the GRCh38 and GRCm38 genomes.
scRNA-sequencing data were annotated using Ensembl version 102.
All other data are available on request from the corresponding author.

Human research participants

Policy information about studies involving human research participants and Sex and Gender in Research.

Reporting on sex and gender	N/A
Population characteristics	N/A
Recruitment	N/A
Ethics oversight	N/A

Note that full information on the approval of the study protocol must also be provided in the manuscript.

Field-specific reporting

Please select the one below that is the best fit for your research. If you are not sure, read the appropriate sections before making your selection.

☒ Life sciences ☐ Behavioural & social sciences ☐ Ecological, evolutionary & environmental sciences

For a reference copy of the document with all sections, see nature.com/documents/nr-reporting-summary-flat.pdf

Life sciences study design

All studies must disclose on these points even when the disclosure is negative.

Sample size	Experimental sample sizes were estimated based on previous experience with derivation of hGPCs and engraftment variability in human glial chimeras. We have extensively published the quantitative data underlying our chimeric model:<br>Human iPSC Glial Mouse Chimeras Reveal Glial Contributions to Schizophrenia, PMID: 28736215<br>Human Glial Progenitor Cells Effectively Remyelinate the Demyelinated Adult Brain, PMID: 32433967<br>Human glia can both induce and rescue aspects of disease phenotype in Huntington disease, PMID: 27273432
Data exclusions	Samples were excluded only when there were technical issues related to experimental procedures as detailed in the methods section of the manuscript and Supplementary Table 2. Otherwise, no data were excluded from this study.
Replication	For experiments regarding the derivation and characterization of hGPCs from engineered reporter GENEA19 and GENEA20 hESCs, each differentiation, starting from a new hESC passage, was considered as an independent biological replicate.<br>For experiments with human glial chimeric mice, experimental transplants were performed once, with transplantations performed on different days using hGPCs derived from independent differentiations. Mice were allocated to experimental groups and timepoints and each hemisphere was considered an independent biological replicate.<br>For all of these experiments, the number of biological replicates are indicated in the figure legends. All attempts at replication were successful.

	The genotyping (Supplementary Figure 2, A) and validation of pluripotency (Supplementary Figure 2, B) of reporter GENEA19 and GENEA20 hESCs were performed once but confirmed by all other experiments (stable expression of fluorescent reporters in human glia derived from these lines upon in vitro differentiation and in vivo maturation).
Randomization	Cell culture experiments (generation of reporter hPSCs and derivation and characterization of reporter hGPCs) were not randomized. For experiments with human glial chimeras, mice were randomly assigned to different experimental groups and timepoints.
Blinding	Investigators were not blinded during generation of reporter hESCs, derivation and characterization of reporter hGPCs and grafting; however, quantitative histology was often performed by investigators/technicians not familiar with the details of the experimental conditions. Blinding was not possible for sequencing based experiments as the identity of samples was apparent from their gene expression.

Reporting for specific materials, systems and methods

We require information from authors about some types of materials, experimental systems and methods used in many studies. Here, indicate whether each material, system or method listed is relevant to your study. If you are not sure if a list item applies to your research, read the appropriate section before selecting a response.

Materials & experimental systems

n/a	Involved in the study
☐	☒ Antibodies
☐	☒ Eukaryotic cell lines
☒	☐ Palaeontology and archaeology
☐	☒ Animals and other organisms
☒	☐ Clinical data
☒	☐ Dual use research of concern

Methods

n/a	Involved in the study
☒	☐ ChIP-seq
☐	☒ Flow cytometry
☒	☐ MRI-based neuroimaging

Antibodies

Antibodies used

Olig2 Mouse 1:200 Millipore MABN50

hGFAP Mouse 1:200 Biolegend SMI-21

hN Mouse 1:200 Abcam ab254080

Ki67 Rabbit 1:200 Invitrogen MA5-14520

EGFP Chicken 1:500 Invitrogen A10262

mCherry Rat 1:500 Invitrogen M11217

PDGFRa Rabbit 1:200 Cell Signalling 5241S

Oct4 Mouse 1:1100 Millipore MAB4401

Rat IgG (H+L) - Alexa Flour 568 Goat 1:400 Invitrogen A-11077

Chicken IgY (H+L) - Alexa Flour Plus 488 Goat 1:400 Invitrogen A32931

Rabbit IgG (H+L) - Alexa Fluor Plus 647 Goat 1:400 Invitrogen A32733

Mouse IgG (H+L) - Alexa Fluor Plus 647 Goat 1:400 Invitrogen A32728

CD140a-FITC Mouse 1:10 BD Horizon 564594

CD140a-PE Mouse 1:10 BD Pharmingen 556002

CD44-APC Mouse 1:500 Miltenyi Biotec 130-113-331

A2B5-APC Mouse 1:50 Miltenyi Biotec 130-093-582

Validation

All antibodies are commercially available and validated by their manufacturers:

Olig2 Mouse 1:200 Millipore MABN50
https://www.emdmillipore.com/US/en/product/Anti-Olig2-Antibody-clone-211F1.1,MM_NF-MABN50

hGFAP Mouse 1:200 Biolegend SMI-21
hhttps://www.biolegend.com/de-at/products/anti-gfap-antibody-11057

hN Mouse 1:200 Abcam ab254080
https://www.abcam.com/nuclei-antibody-235-1-ab254080.html

Ki67 Rabbit 1:200 Invitrogen MA5-14520
https://www.thermofisher.com/antibody/product/Ki-67-Antibody-clone-SP6-Recombinant-Monoclonal/MA5-14520

EGFP Chicken 1:500 Invitrogen A10262
https://www.thermofisher.com/antibody/product/GFP-Antibody-Polyclonal/A10262

mCherry Rat 1:500 Invitrogen M11217
https://www.thermofisher.com/antibody/product/mCherry-Antibody-clone-16D7-Monoclonal/M11217

PDGFRa Rabbit 1:200 Cell Signalling 5241S
https://www.cellsignal.com/products/primary-antibodies/pdgf-receptor-a-d13c6-xp-rabbit-mab/5241

Oct4 Mouse 1:1100 Millipore MAB4401
https://www.emdmillipore.com/US/en/product/Anti-Oct-4-clone-10H11.2-100g-KC-Antibody,MM_NF-MAB4401-CKC

Rat IgG (H+L) - Alexa Flour 568 Goat 1:400 Invitrogen A-11077
https://www.thermofisher.com/antibody/product/Goat-anti-Rat-IgG-H-L-Cross-Adsorbed-Secondary-Antibody-Polyclonal/A-11077

Chicken IgY (H+L) - Alexa Flour Plus 488 Goat 1:400 Invitrogen A32931
https://www.thermofisher.com/antibody/product/Goat-anti-Chicken-IgY-H-L-Cross-Adsorbed-Secondary-Antibody-Polyclonal/A32931

Rabbit IgG (H+L) - Alexa Fluor Plus 647 Goat 1:400 Invitrogen A32733
https://www.thermofisher.com/antibody/product/Goat-anti-Rabbit-IgG-H-L-Highly-Cross-Adsorbed-Secondary-Antibody-Polyclonal/A32733

Mouse IgG (H+L) - Alexa Fluor Plus 647 Goat 1:400 Invitrogen A32728
https://www.thermofisher.com/antibody/product/Goat-anti-Mouse-IgG-H-L-Highly-Cross-Adsorbed-Secondary-Antibody-Polyclonal/A32728

CD140a-FITC Mouse 1:10 BD Horizon 564594
https://www.bdbiosciences.com/en-us/products/reagents/flow-cytometry-reagents/research-reagents/single-color-antibodies-ruo/bb515-mouse-anti-human-cd140a.564594

CD140a-PE Mouse 1:10 BD Pharmingen 556002
https://www.bdbiosciences.com/en-us/products/reagents/flow-cytometry-reagents/research-reagents/single-color-antibodies-ruo/pe-mouse-anti-human-cd140a.556002

CD44-APC Mouse 1:500 Miltenyi Biotec 130-113-331
https://www.miltenyibiotec.com/US-en/products/cd44-antibody-anti-human-db105.html#pe-vio-770:100-tests-in-200-ul

A2B5-APC Mouse 1:50 Miltenyi Biotec 130-093-582
https://www.miltenyibiotec.com/US-en/products/a2b5-antibody-anti-human-mouse-rat-105hb29.html#apc:30-tests-in-60-ul

Eukaryotic cell lines

Policy information about cell lines and Sex and Gender in Research

Cell line source(s)	GENEA019 and GENEA020 are both female ESC lines obtained from GENEA.
Authentication	These lines were not authenticated
Mycoplasma contamination	All cell lines tested negative for mycoplasma.
Commonly misidentified lines (See ICLAC register)	No commonly misidentified lines were used.

Animals and other research organisms

Policy information about studies involving animals; ARRIVE guidelines recommended for reporting animal research, and Sex and Gender in Research

Laboratory animals	Immunodeficient Rag1 null mice were utilized between postnatal day 1 and 72 weeks.
Wild animals	No wild animals were used in this study
Reporting on sex	Both sexes of mice were used in this study

Field-collected samples: No field-collected samples were used in this study

Ethics oversight: All experiments were approved by the Institutional Animal Care and Use Committee of the University of Rochester

Note that full information on the approval of the study protocol must also be provided in the manuscript.

Flow Cytometry

Plots

Confirm that:

☒ The axis labels state the marker and fluorochrome used (e.g. CD4-FITC).

☒ The axis scales are clearly visible. Include numbers along axes only for bottom left plot of group (a 'group' is an analysis of identical markers).

☒ All plots are contour plots with outliers or pseudocolor plots.

☒ A numerical value for number of cells or percentage (with statistics) is provided.

Methodology

Sample preparation

In vitro flow cytometry of human GPCs:
Glial cultures were collected as a single cell suspension following 5 min dissociation in Accutase, counted with a hemocytometer, and resuspended at 106 cell/ml in Miltenyi wash buffer (MWB; PBS + 0.5% BSA Fraction V (ThermoFisher cat. no. 15260037) + 2 µM EDTA (ThermoFisher cat. no. 15575020)). Each cell suspension was then incubated in MWB for 15 mins at 4ºC to block non-specific antibody binding and divided in 100 µL fractions for immunolabelling. Each fraction was then incubated with fluorophore-conjugated antibodies for 15 min at 4°C, except for the unstained gating controls. Antibody sources and concentrations are listed in Supplementary Table 1. Cells were then washed with MWB, spun for 10 min at 200 x g, resuspended in MWB, and strained into 5 ml polystyrene tubes with 35 µm cell-strainer caps (Corning, cat. no. 352235). To exclude dead cells, 4′,6-diamidino-2-phenylindole (DAPI; ThermoFisher cat. no. D1306) was added at 1 µg/mL. Flow cytometry analysis of glial cultures was then performed on a CytoFLEX S platform (Beckman Coulter), and the data analysed with the CytExpert (Beckman Coulter) and FlowJo (BD Biosciences) software. Gating strategy and data analysis are exemplified in Supplementary Fig. 4.

scRNA-seq of human glial chimeric mice:
Mice were euthanized with euthasol, transcardially perfused with sterile Hank's Balanced Salt Solution (HBSS) containing magnesium chloride and calcium chloride, and the brain removed. The brains were immersed in ice-cold sterile HBSS for about 5 minutes to facilitate the microdissection. Under a dissecting microscope, the striata from each mouse was dissected and placed in sterile HBSS on ice. The striatal tissues were transferred to a Petri dish containing sterile HBSS without magnesium chloride and calcium chloride, chopped into small pieces using sterile disposable scalpels, transferred into a sterile tube, and then incubated in a papain/DNase dissociation solution at 37°C for 50 minutes. Ovomuccoid dissolved in EBSS was then added to inactivate the papain. The tissue was triturated by repeated pipetting in order to achieve a single cell suspension. The cells were then pelleted, resuspended into MEM, and filtered trained into 5 ml polystyrene tubes with 35 µm cell-strainer caps (Corning, cat. no. 352235). To exclude dead cells, 4′,6-diamidino-2-phenylindole (DAPI; ThermoFisher cat. no. D1306) was added at 1 µg/mL for FACS. Single cell preparations were isolated based on their expression of mCherry, EGFP, or their absence, using a BD FACSAria Fusion (BD Biosciences, BD FACS Diva software). Our gating strategy is shown in Supplementary Fig. 7B.

Instrument: Beckman Coulter CytoFLEX for in vitro flow cytometry. BD Aria Fusion for human glial chimeric FACS.

Software: BD FACS Diva, CytExpert, and FlowJo

Cell population abundance: The abundance of populations was determined via scRNA-seq.

Gating strategy: FCS and SSC gates were adjusted to isolate whole cells. Gating of fluorescent cells in singly engrafted mice were drawn against unengrafted mouse striatum. These gates were then used in co-engrafted mice for two color sorts.

☒ Tick this box to confirm that a figure exemplifying the gating strategy is provided in the Supplementary Information.
